# Supplementary material for: Orientated crystallization of FA-based perovskite via hydrogen-bonded polymer network for efficient and stable solar cells
Source: Nat Commun. 2023 Feb 2;14:573. doi: 10.1038/s41467-023-36224-6 (PMC9895431; doi:10.1038/s41467-023-36224-6)
Supplement: Supplementary file 1 — Supplementary Information [file 41467_2023_36224_MOESM1_ESM.pdf]

# Supplementary Information

## Orientated crystallization of FA-based perovskite via hydrogen-bonded polymer network for efficient and stable solar cells

Li et al.

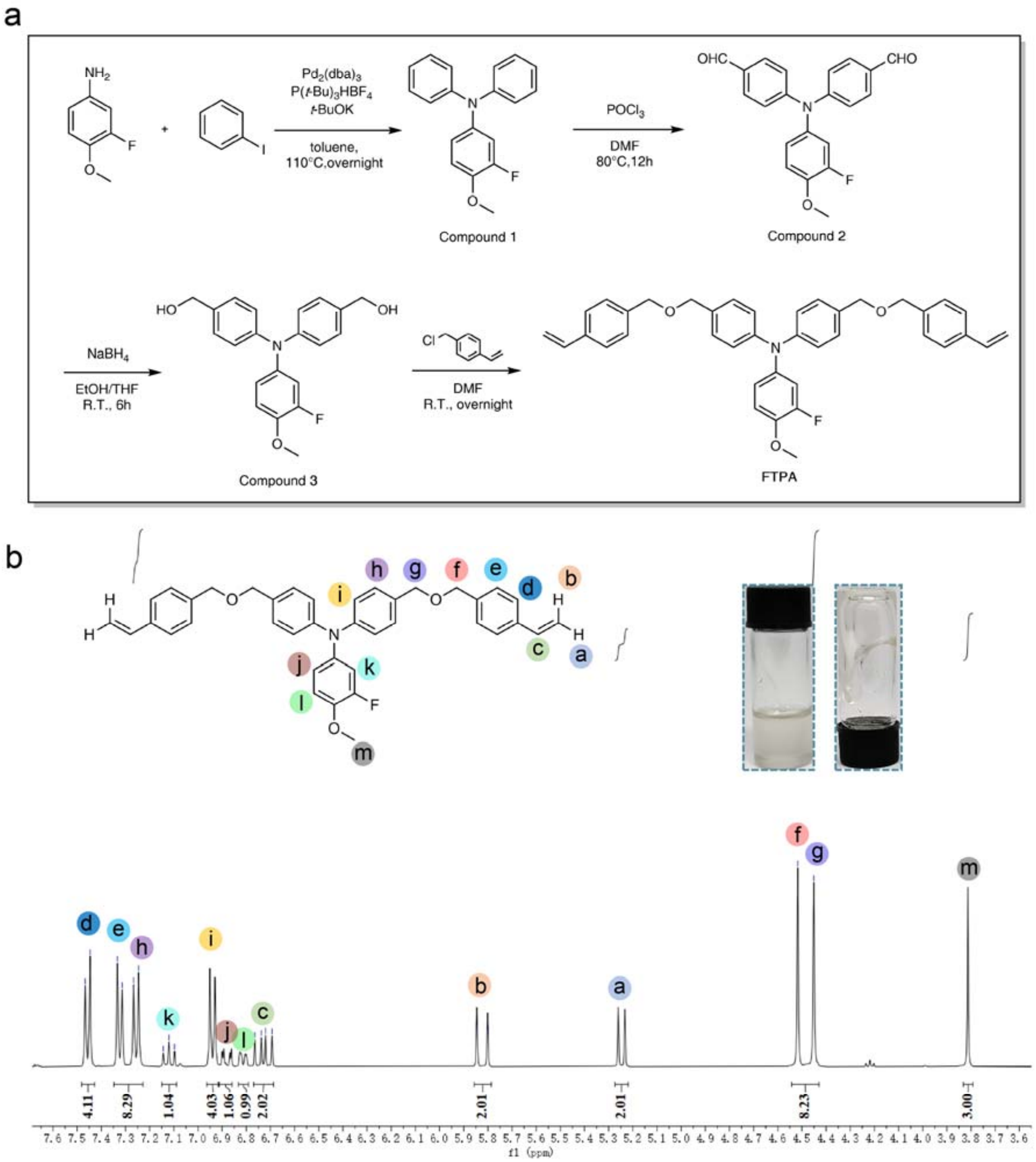


**Supplementary Fig. 1.** (a) Synthetic route and (b) $^1$HNMR spectrum of FTPA. The inert photographs show that the pure FTPA is a viscous liquid.

**Supplementary Note 1**

Synthesis procedures of FTPA:

Synthesis of 3-fluoro-4-methoxy-N,N-diphenylaniline (Compound **1**) proceeds as follows. In a 150mL Schrank bottle, 3-fluoro-4-methoxyaniline (2.5 g, 17.7 mmol), Iodobenzene (7.59 g, 37.2 mmol), $Pd_2(dba)_3$ (0.48 g, 0.531 mmol), *t*-BuOK (6.0 g, 53.1 mmol) and P(*t*-Bu)$_3$HBF$_4$ (0.50 mg, 1.77 mmol) were dissolved in 60 mL of ortho-xylene. After pumping three times, the reaction was carried out under an argon atmosphere, heated to 110 °C, and reacted overnight. Water was added to the mixture, which was then extracted with dichloromethane. The organic layer was dried with $Na_2SO_4$ and concentrated in vacuo. The crude product was purified by silica gel column chromatography (dichloromethane/ petroleum ether 1/8) to afford the title compound as a white solid (4.20 g, 83%). $^1$H-NMR (400MHz, Chloroform-d) δ(ppm) 7.15(d, *J*=7.3Hz, 4H), 6.97(dd, *J*=8.7, 1.1Hz, 4H), 6.92(t, *J*=7.3Hz, 2H), 6.84 – 6.80(m, 1H), 6.79(d, *J*=2.3Hz, 1H), 6.76(t, *J*=3.0Hz, 1H), 3.80(s, 3H). $^{13}$C-NMR (101MHz, Chloroform-d) δ(ppm) 155.15, 151.54, 147.77, 144.96, 141.48, 129.34, 123.62, 122.28, 121.17, 115.04, 112.68, 57.42. MS: m/z (%) $[M+H]^+$ calcd for $C_{19}H_{16}FNO$: 293.121; found: 293.121.

Synthesis of 4,4'-((3-fluoro-4-methoxyphenyl) azanediyl) dibenzaldehyde (Compound **2**). In a Schlenk flask (25 mL), 3-fluoro-4-methoxy-N,N-diphenylaniline (1.0 g, 3.41 mmol) and DMF (6 mL) were mixed.Then, $POCl_3$ (3 mL) was added dropwise in an ice bath. Reflux the mixture for 12 h under argon atmosphere. The mixture was cooled to room temperature and quenched with cold water (100 mL). Extract the crude product with dichloromethane. The organic phase was dried over sodium sulfate. The solvent was removed under reduced pressure and the crude product was purified by silica gel flash column chromatography (ethyl acetate/petroleum ether 1/4) to afford the product as a yellow solid. (0.91 g, 76%). $^1$H-NMR (400MHz, DMSO-d6) δ(ppm) 9.87 (s, 2H), 7.83 (d, *J*=8.6Hz, 4H), 7.26 (t, *J*=9.2Hz, 1H), 7.20 (d, *J*=2.6Hz, 1H), 7.16 (d, *J*=8.6Hz, 4H), 7.04 (d, *J*=5.8Hz, 1H), 3.87 (s, 3H). $^{13}$C-NMR (101MHz, DMSO-D6) δ(ppm) 191.74, 153.61, 151.76, 151.15, 146.54, 146.43, 138.06, 137.98, 131.83, 131.40, 124.73, 124.70, 122.51, 116.24, 116.05, 115.51, 115.48, 56.68. MS: m/z (%)$[M+H]^+$ calcd for $C_{21}H_{16}FNO_3$: 349.111; found: 349.112.

Synthesis of (((3-fluoro-4-methoxyphenyl)azanediyl) bis(4,1-phenylene)) dimethanol (Compound **3**). To a solution of compound 2 (1.0 g, 2.86 mmol) in EtOH: THF=1:1 (30 mL) was slowly added $NaBH_4$ (0.34 g, 8.58 mmol). The mixture was stirred under $N_2$ for 6 hours for the reaction. At the end of the monitored reaction, 20 mL of water was added to quench the reaction and the organic phase was extracted with ethyl acetate after removal of the solvent, the residue proceeded directly to the next step without passing through purification to give the crude compound 3 (0.95 g, 95%). $^1$H-NMR (400MHz, DMSO-$d_6$) δ(ppm) 7.21 (d, *J*=8.5 Hz, 4H), 7.14–7.05 (m, 1H), 6.90 (d, *J*=8.4 Hz, 4H), 6.80 (d, *J*=2.6Hz, 1H), 6.79–6.71 (m, 1H), 5.11 (t, *J*=5.7Hz, 2H), 4.41 (d, *J*=5.6Hz, 4H), 3.79 (s, 1H). $^{13}$C-NMR (101 MHz, Chloroform-d) δ(ppm) 155.30, 151.52, 147.22, 144.96, 141.20, 135.14, 127.97, 123.55, 120.71, 114.22, 113.41, 65.05, 60.56, 56.71, 21.17, 14.29. MS: m/z (%) $[M+H]^+$ calcd for $C_{21}H_{20}FNO_3$: 353.147; found: 353.147.

Synthesis of 3-fluoro-4-methoxy-4,4'-bis((4-vinylbenzyl ether) methyl)) triphenylamine (FTPA). To a solution of compound 3 (2.96 g, 8.38 mmol) in DMF (50 mL) was slowly added sodium hydride(0.86 g, 35.19 mmol) for 1 hour at room temperature. 4-vinylbenzyl chloride (5.45 g, 35.19 mmol) was then added to the solution and the mixture was stirred for 12 hours. At the end of the reaction, water was added to quench the reaction and the reaction mixture would be extracted withdichloromethane and water. The organic layer is dried over anhydrous $Na_2SO_4$ and filtered. The product was separated by silica gel column chromatography. Elution using petroleum ether:ethyl acetate (15:1) gave colorless and clear oily substance (2.30g, 46%). $^1$H-NMR (400MHz, DMSO-$d_6$,) δ(ppm) 7.41 (d, J=8.2Hz, 4H), 7.28 (d, *J*=8.3Hz, 4H), 7.21 (d, *J*=8.5Hz, 4H), 7.11–7.04 (m, 1H), 6.89 (d, *J* =8.5Hz, 4H), 6.83 (dd, *J*=12.8, 2.6Hz, 1H), 6.77 (d, *J*=8.8Hz, 1H), 6.68 (dd, *J*=17.7, 11.0Hz, 2H), 5.78 (d, *J*=18.7Hz, 2H), 5.20 (d, *J*=11.9Hz, 2H), 4.44 (d, *J*=26.6Hz, 8H), 3.77 (s, 3H). $^{13}$C-NMR (101 MHz, DMSO-$D_6$) δ(ppm) 147.05, 144.24, 138.71, 136.93, 136.84, 133.02, 129.58, 128.34, 126.61, 123.20, 121.74, 115.28, 114.68, 113.63, 113.43, 71.65, 56.69. HRMS (APCI) for: $C_{39}H_{36}F_1N_1O_3$ $[M+H]^+$: calcd: 585.267, found: 585.267

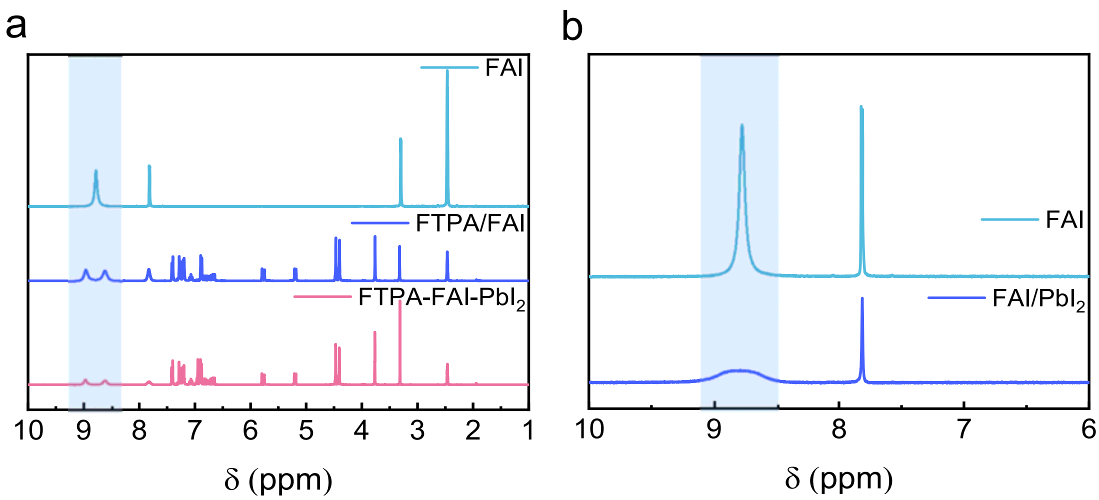


**Supplementary Fig. 2.** Investigation of interactions between FTPA and the component of perovskite. $^{1}$H NMR spectra of (a) FAI, FTPA/FAI, and FTPA/FAI/$PbI_2$, (b) FAI and FAI/$PbI_2$.

**Supplementary Note 2:** As shown in **Supplementary Fig. 2b**, if only addition of $PbI_2$ in FAI, the resonance signal of the ammonium decreases in intensity, but does not split. This indicates that the interaction of $FA^+$ and FTPA was stronger than $FA^+$ with $PbI_2$. (See discussion in **Fig. 1b**).

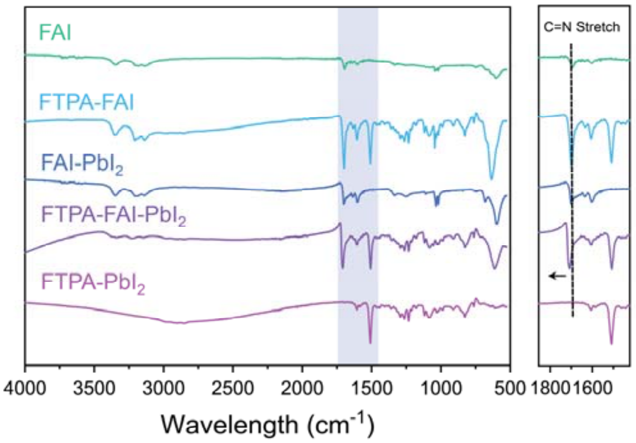


**Supplementary Fig. 3.** Fourier Transform Infrared Spectroscopy (FTIR) spectra show the interaction between FTPA and the component of perovskite. The shift VC=N can be attributed to the hydrogen-bonded interaction between C=NH and FTPA.

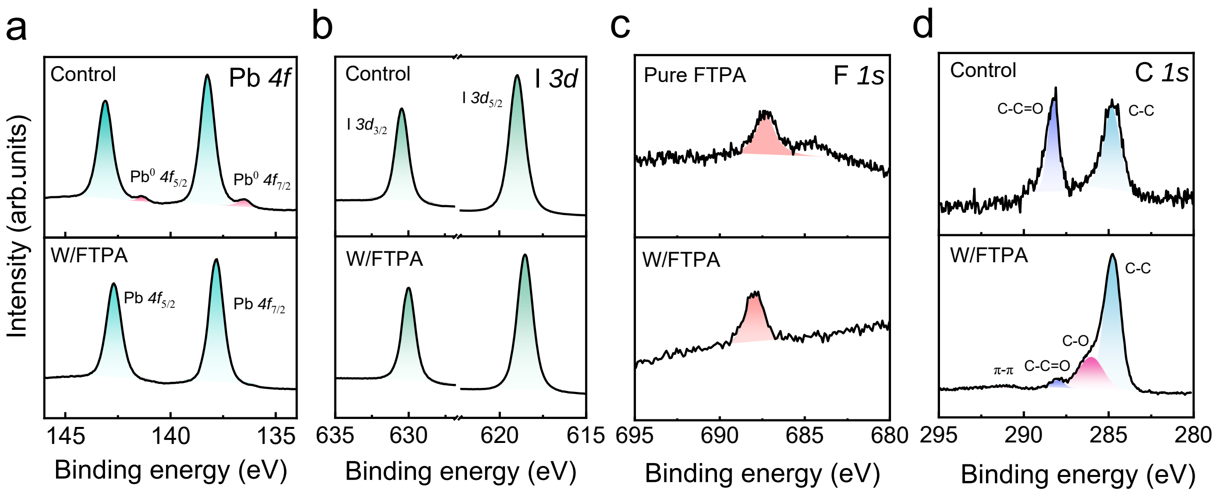


**Supplementary Fig. 4.** XPS spectra of the Control perovskite films, pure FTPA and perovskite films prepared with FTPA as additive. (a) Pb *4f*, (b) I *3d*, (c) F *1s*, (d) C *1s*.

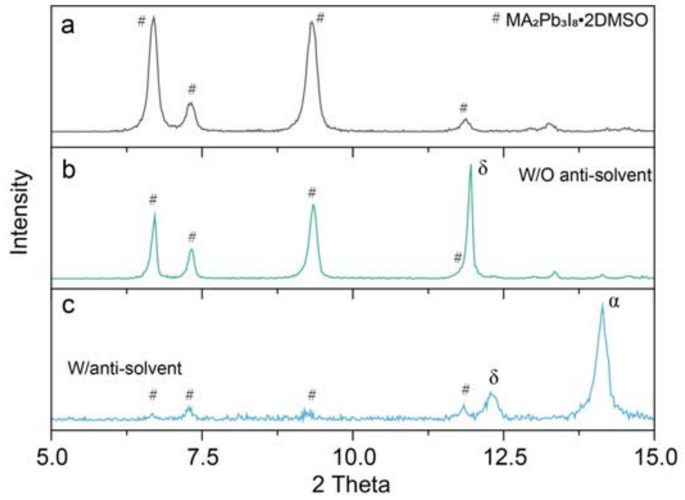


**Supplementary Fig. 5.** X-ray diffraction (XRD) patterns of the wet perovskite film. (a) Perovskite film fabricated using perovskite precursors consisting of MAI: $PbI_2$ (1:1, mol%) in DMF: DMSO (8:1, v/v). (b) Control perovskite film without anti-solvent during spin-coating. (c) Control perovskite film with anti-solvent during spin-coating.

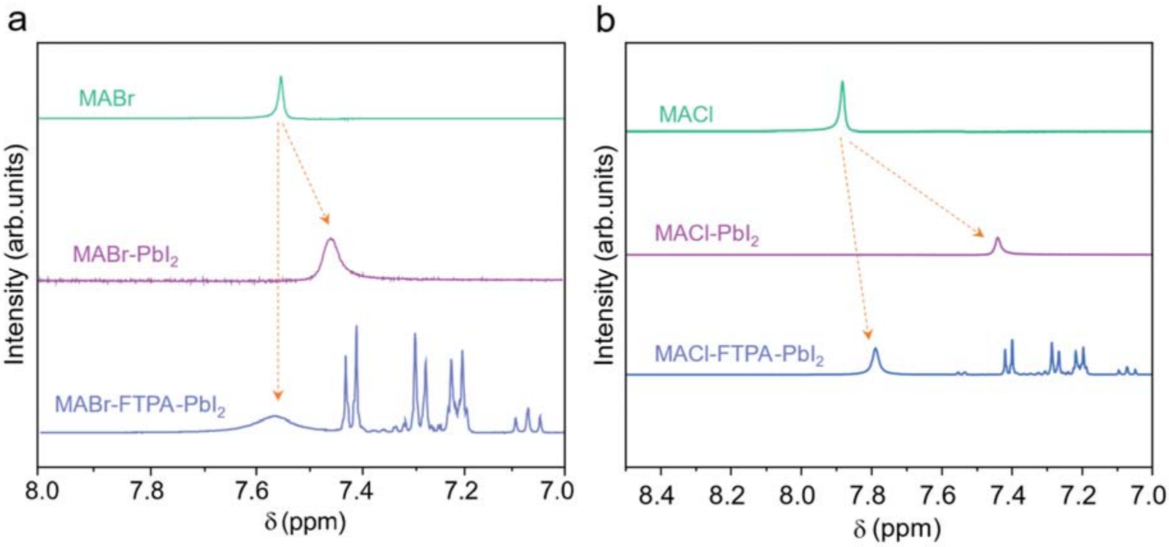


**Supplementary Fig. 6.** Investigation of interactions between FTPA and the component of perovskite (MACl, MABr, and $PbI_2$). 1H NMR spectra of (a) MACl, MACl/$PbI_2$ (1:1, mol%), and MACl/$PbI_2$/FTPA (1:1:0.5, mol%). (b) MABr, MABr/$PbI_2$ (1:1, mol%), and MABr/$PbI_2$/FTPA (1:1:0.5, mol%).

**Supplementary Note 3:** As shown in **Supplementary Fig. 6a**, in the pure deuterated DMSO solution, the resonance signal of protonated ammonium ($MA^+$) of MABr was at 7.539 ppm. With the addition of $PbI_2$, the resonance signal of $MA^+$ showed a large shift toward high field to 7.439 ppm, implying that the strong interaction between $MA^+$ and $PbI_2$. After adding FTPA into this solution, the $MA^+$ signal remained unchanged. In **Supplementary Fig. 6b,** the resonance signal of $MA^+$ of MACl was at 7.883 ppm. With the addition of $PbI_2$, the resonance signal of the $MA^+$ showed a large shift toward high field to 7.441 ppm, implying that the strong interaction between $MA^+$ and $PbI_2$. After adding FTPA into this solution, the $MA^+$ signal becomes less susceptible to $PbI_2$, and shifted back to the low field to 7.790 ppm. These results imply that FTPA suppressed the interaction between $MA^+$ and $PbI_2$, thereby inhibited the formation of intermediate phase $MA_2Pb_3I_8\cdot 2DMSO$ from the source.

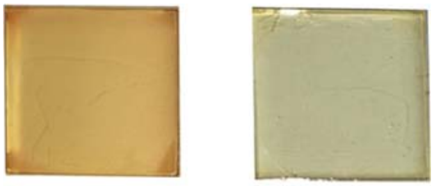


**Supplementary Fig. 7.** Photographs of the wet perovskite film after spin-coating without using antisolvent.

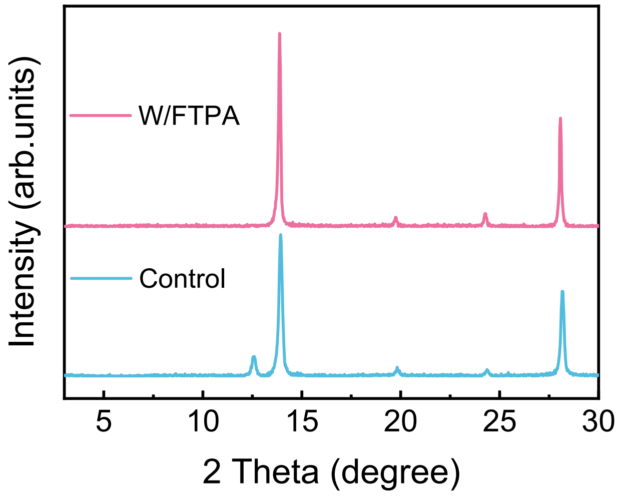


**Supplementary Fig. 8.** X-ray diffraction of the Control and perovskite films with FTPA after annealing at 100 °C for 1 h.

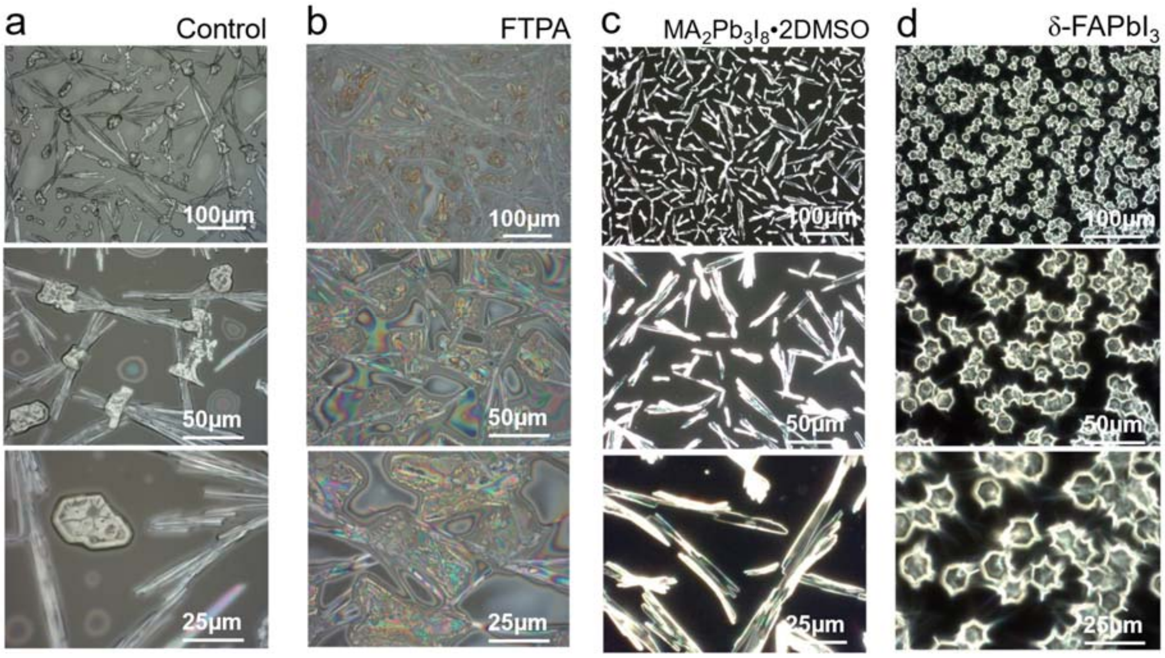


**Supplementary Fig. 9.** Microscope images of different wet perovskite films after spin-coating process without using antisolvent: (a) Control perovskite consisting of $FA_{0.95}MA_{0.05}Pb(I_{0.95}Br_{0.05})_3$, (b) perovskite with FTPA as additive, (c) $MA_2Pb_3I_8$·2DMSO intermediate phase prepared by perovskite of $MAI/PbI_2$ (1:1, mol%), (d) δ-$FAPbI_3$ prepared by perovskite of $FAI/PbI_2$ (1:1, mol%).

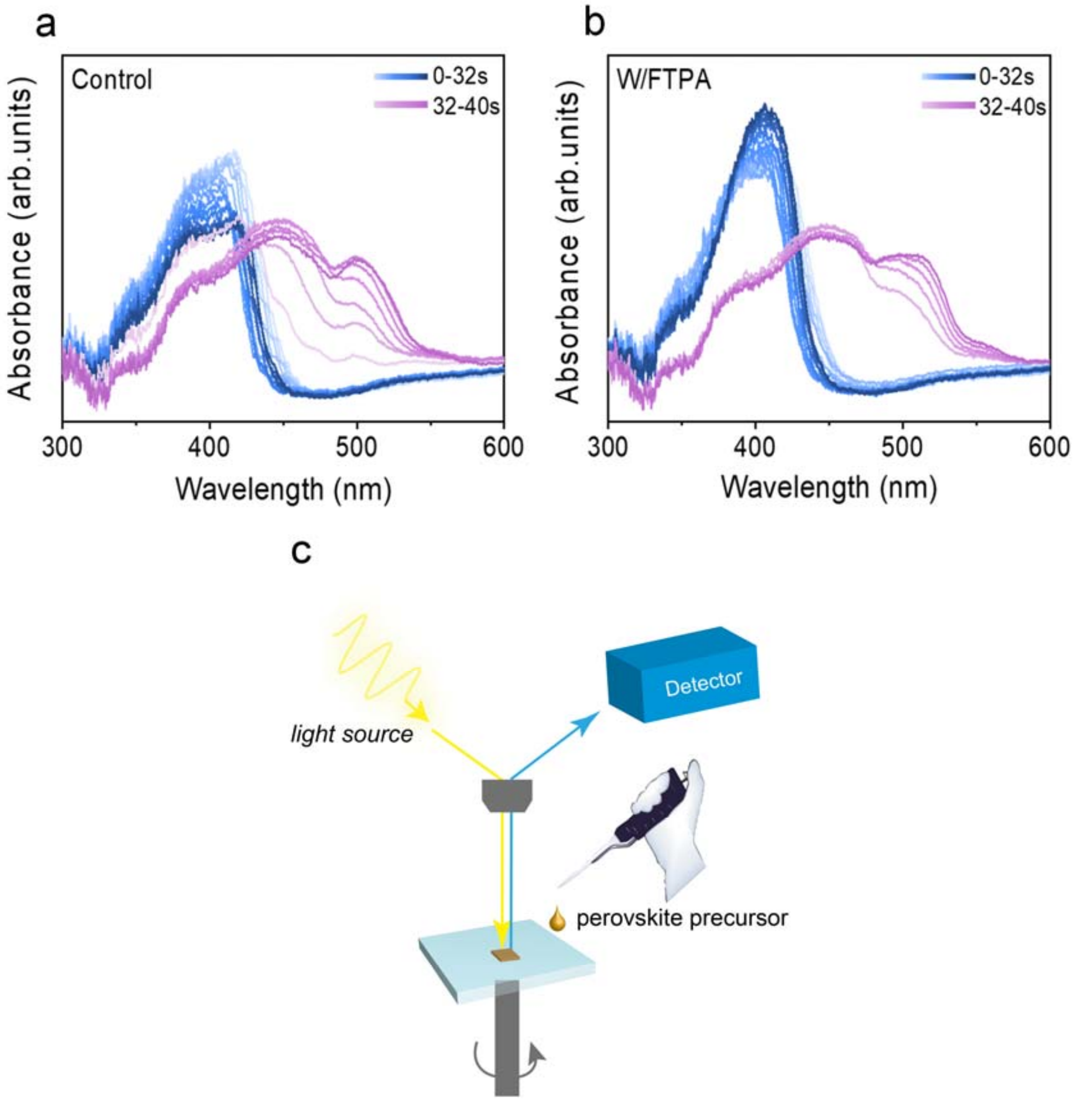


**Supplementary Fig. 10.** In-situ UV-vis absorption of perovskite films during spin-coating process (0-40 s). (a) Control perovskite and (b) perovskite treated with FTPA as additive. (c) Schematic of the in-situ UV-vis absorption measurement consisting of light source, detector, and a spin-coater.

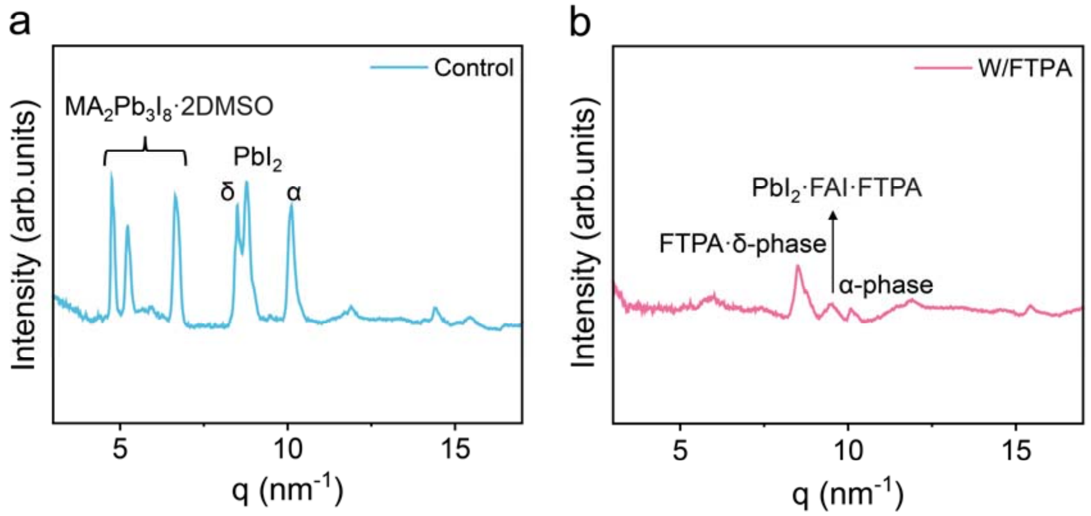


**Supplementary Fig. 11.** In-situ GIWAXS intensity profiles for the perovskite films during spin-coating process (a) Control perovskite and (b) perovskite with FTPA as additive.

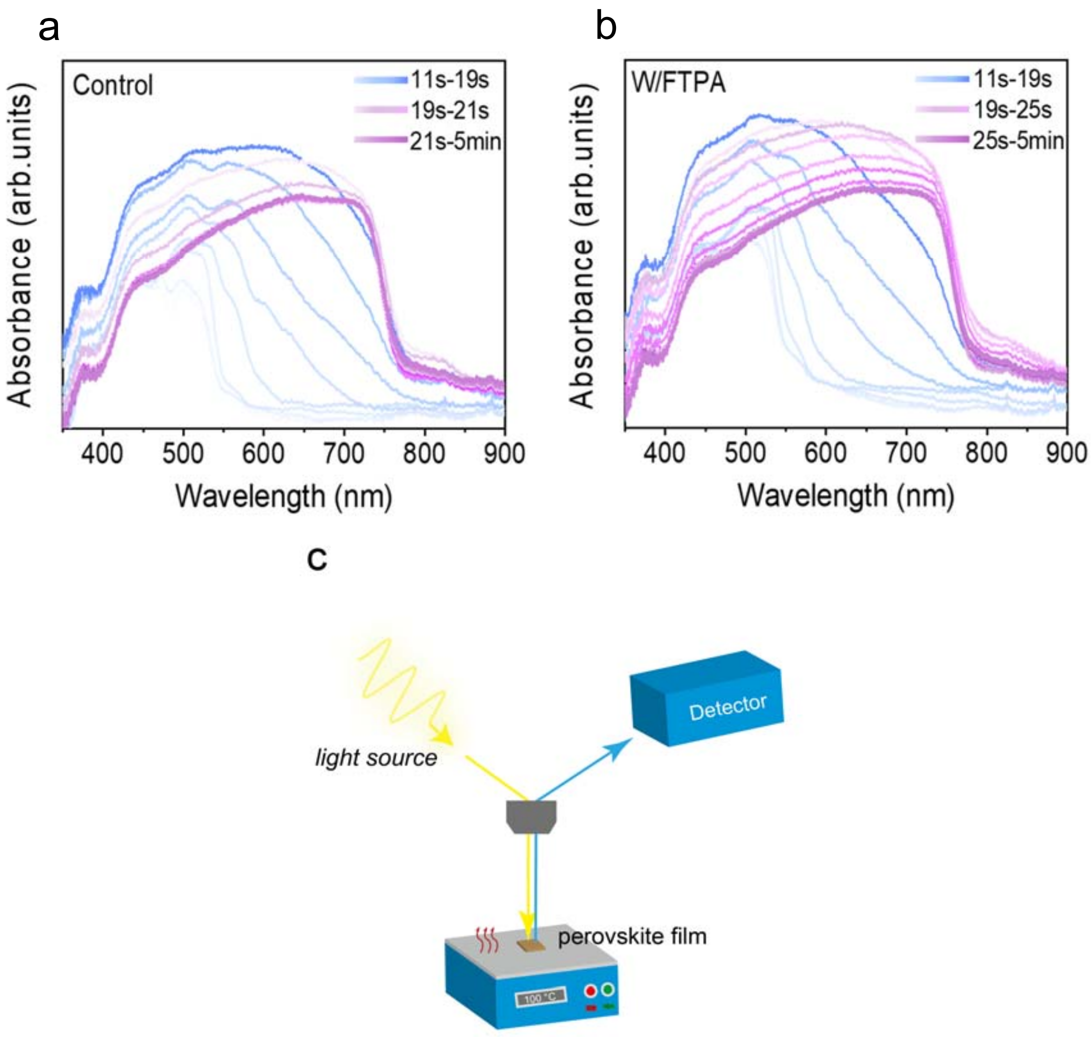


**Supplementary Fig. 12.** In-situ UV-vis absorptions for the perovskite films during spin-coating process (a) Control perovskite and (b) perovskite with FTPA as additive. (c) Schematic of the in-situ UV-vis absorption measurement consisting of light source, detector, and a hot plate.

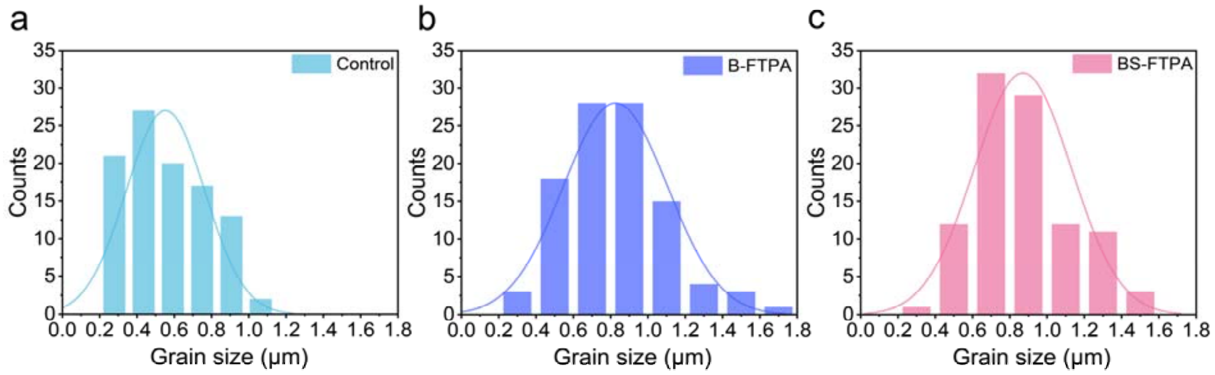


**Supplementary Fig. 13.** Distribution histogram of crystal grain sizes of the perovskite thin films: (a) Control, (b) B-FTPA and (c) BS-FTPA, respectively.

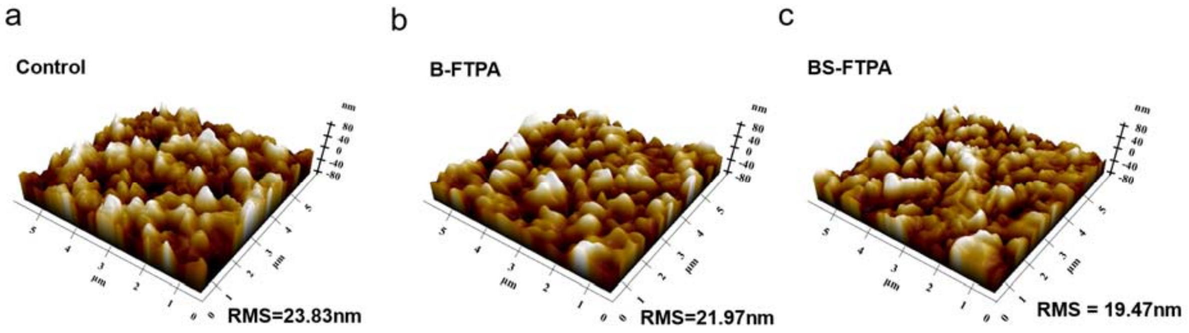


**Supplementary Fig. 14.** Atomic force microscopy (AFM) surface images and root-mean-square (RMS) roughness of (a) Control perovskite, (b) B-FTPA, (c) BS-FTPA.

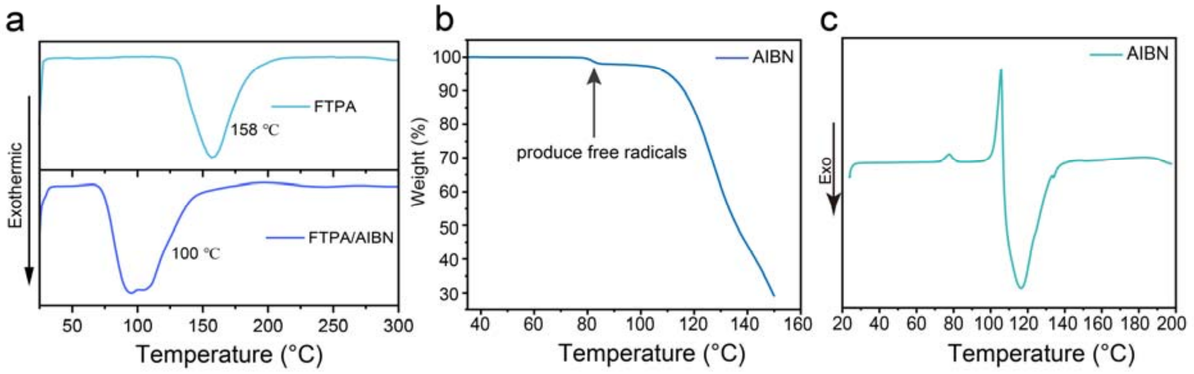


**Supplementary Fig. 15.** (a) Differential Scanning Calorimetry (DSC) thermograms of FTPA and FTPA with the free radical initiator of 2,2′-azobis-isobutyronitrile (AIBN, 0.1 mol%). (b) TGA curves of AIBN. (c) DSC curves of AIBN.

**Supplementary Note 4:** AIBN was used as a free radical initiator in the perovskite precursor to help FTPA convert into cross-linked polymer via atom transfer radical polymerization (ATRP) during perovskite annealing-process. For the B-FTPA system, the molar concentration of perovskite is 1.474 mmol, the doping ratio of FTPA was 5 mol% of perovskite about 0.073 mmol in the perovskite precursor. AIBN is only 0.1 mol% of FTPA about $0.73\times10^{-4}$ mmol in the perovskite precursor, which is one ten thousandth of the perovskite content. Compared to the perovskite precursor and FTPA, the addition of trace amounts of AIBN does not affect the crystallization kinetics of perovskite. In addition, the thermal decomposition temperature of AIBN measured by Thermogravimetric analysis (TGA) (**Supplementary Fig. 15b**) is between 75 and 85 °C to produce free radicals, and then fast decompose after 100 °C (the annealing temperature of perovskite), thus does not remain in the final perovskite films. The differential scanning calorimeter (DSC) (**Supplementary Fig. 15c**) was also confirmed the thermal process as reported in the Ref. 1-3

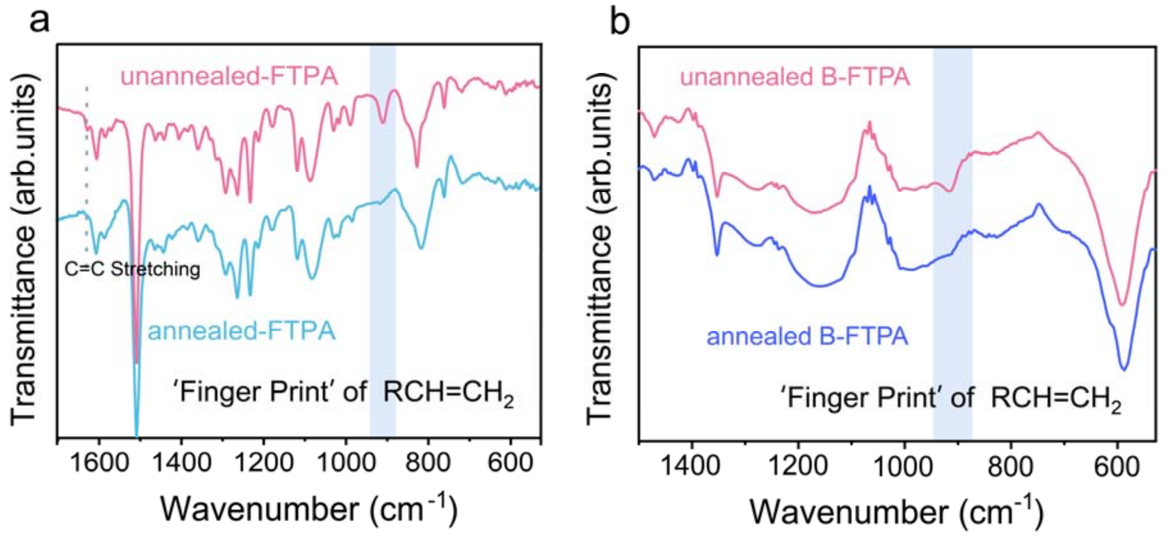


**Supplementary Fig. 16.** Fourier Transform Infrared Spectroscopy (FTIR) spectroscopy of (a) FTPA films and (b) B-FTPA films before and after annealing. The blue square highlights the stretching vibration of $RCH=CH_2$ bonds.

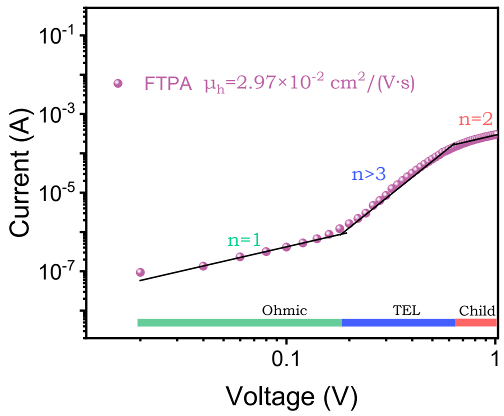


**Supplementary Fig. 17.** *J-V* characteristics of hole-only devices based on pure FTPA.

**Supplementary Note 5:**

SCLC measurement of FTPA: FTO/PEDOT:PSS/FTPA/Spiro-OMeTAD/Au

SCLC measurement of perovskite: hole-only devices with following structures: FTO/PEDOT:PSS/perovskite/Spiro-OMeTAD/Au and electron-only devices with following structures: FTO/CBD-$SnO_2$/perovskite/$PC_{61}BM$/Ag.

The hole/electronic mobility was derived by fitting *J–V* curve with Supplementary Equation (1):

$$J = \frac{9}{8}\varepsilon_r\varepsilon_0\mu_{h/e}\frac{V^2}{L^3} \tag{1}$$

Where $J$ is the current density. $\varepsilon_0$ is the vacuum permittivity. $\varepsilon_r$ is the relative dielectric constant of FTPA or perovskite. $\mu_{h/e}$ is the hole/electronic mobility. $L$ is the thickness of FTPA layer or perovskite layer. $V$ is the applied voltage.

Trap densities ($N_t$) of devices were according to Supplementary Equation (2):

$$N_t = 2\varepsilon_r\varepsilon_0\frac{V_{TFL}}{eL^2} \tag{2}$$

Where $V_{TFL}$ is the voltage at which traps are filled, $e$ is the elementary charge, $L$ is the perovskite layer thickness, $\varepsilon_0$ is the vacuum permittivity, and $\varepsilon_r$ is the dielectric constant.

**Supplementary Table 1.** Parameters of the time-correlated single photon counting (TCSPC) spectroscopy of perovskite films, respectively.

| | $\tau_1$[ns] | $A_1$[%] | $\tau_2$[ns] | $A_2$[%] | $\tau_{ave}$[ns] |
|---|---|---|---|---|---|
| B-FTPA | 27.1 | 16.2 | 325.1 | 83.8 | 320.4 |
| Control | 25.4 | 27.9 | 282.3 | 72.1 | 273.6 |
| BS-FTPA | 7.2 | 42.6 | 77.4 | 57.4 | 50.1 |
| BS-FTPA/Spiro-OMeTAD | 4.2 | 53.4 | 48.9 | 46.6 | 26.9 |

The PL decay time and amplitudes are modeled using Supplementary Equation (3):

$$f(t) = \sum_i A_i\, exp\left(-\frac{t}{\tau_i}\right) + K \tag{3}$$

where $A_i$ is the decay amplitude, $\tau_i$ is the decay time and $K$ is a constant for the base-line offset. The average PL decay times ($\tau_{ave}$) are further estimated using the $\tau_i$ and $A_i$ values from the fitted curve data Extended Data Table 1 using Supplementary Equation (4):

$$\tau_{ave} = \frac{\sum A_i \tau_i^2}{\sum A_i\, \tau_i} \tag{4}$$

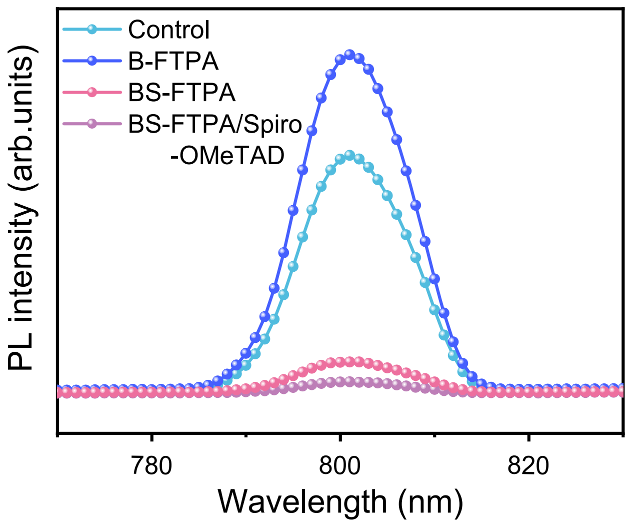


**Supplementary Fig. 18.** Photoluminescence spectra for the Control perovskite, B-FTPA, BS-FTPA and BS-FTPA/Spiro-OMeTAD.

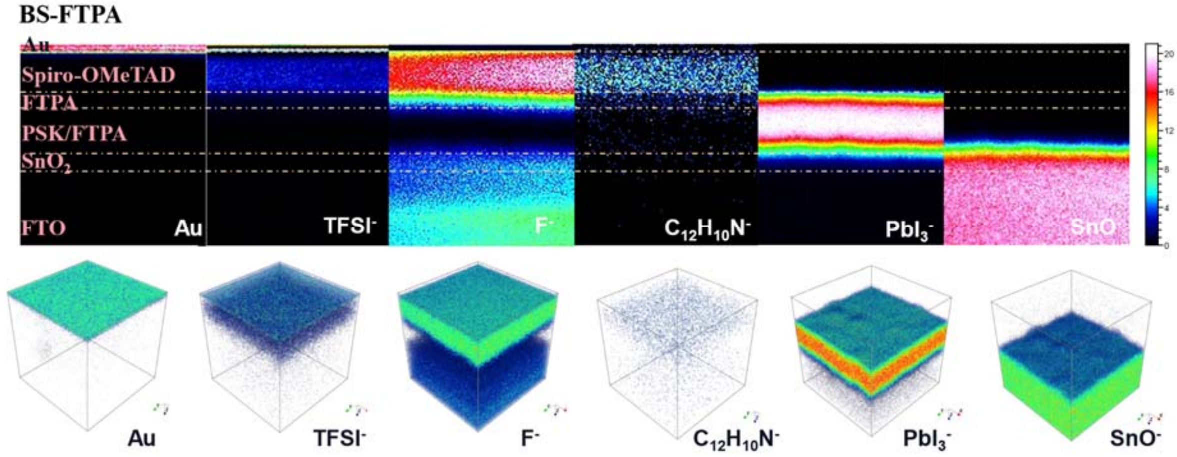


**Supplementary Fig. 19.** ToF-SIMS cross-section image and three-dimensional image of BS-FTPA perovskite device in negative ion model with Au, $TFSI^-$, $F^-$, $C_{12}H_{10}N^-$, $PbI_3^-$ and $SnO^-$.

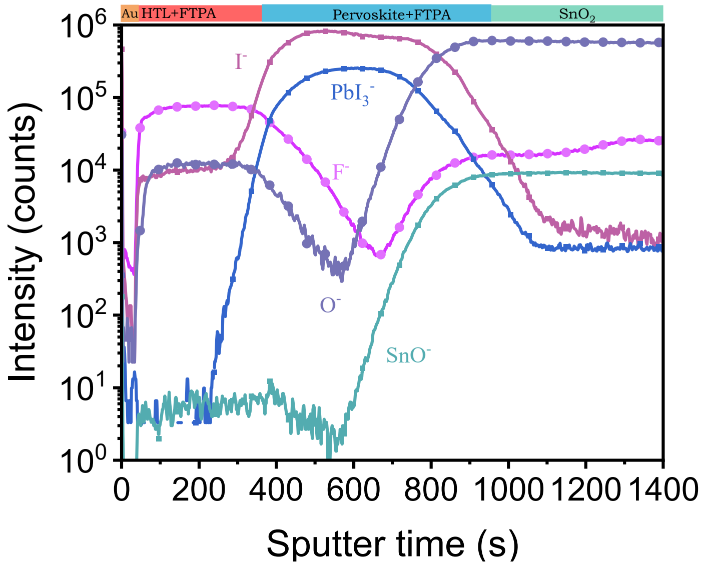


**Supplementary Fig. 20.** ToF-SIMS depth profile of the BS-FTPA perovskite device in negative ion model.

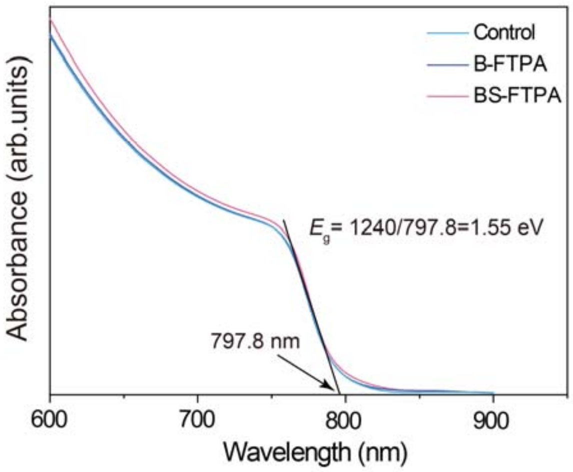


**Supplementary Fig. 21.** UV-vis absorption spectra of the Control, B-FTPA and BS-FTPA perovskite films.

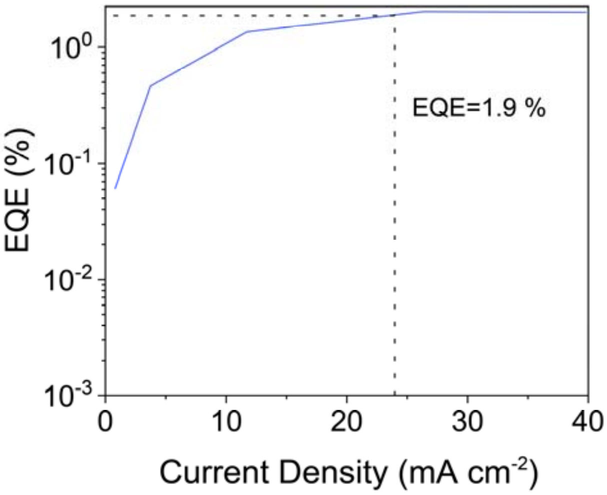


**Supplementary Fig. 22.** Plot of external electroluminescence quantum efficiency ($EQE_{EL}$) versus current density. We quantified the nonradiative recombination losses according to the external quantum efficiency (EQE) of electroluminescence (EL) by operating a solar cell. The $EQE_{EL}$=1.9% is chosen at an injection current that is equal to the photocurrent of 23.76 mA cm$^{-2}$. We estimate the nonradiative losses ($\Delta V_{OC,nonrad}$) of the photo-voltage according to the Supplementary Equation (5):

$$\Delta V_{OC,nonrad} = \frac{kT}{e} \ln EQE_{EL} \tag{5}$$

where $k$ is Boltzmann constant, $T$ is the temperature, and $e$ is the elementary charge. The $\Delta V_{OC,nonrad}$ is calculated to be ≈0.108 V.

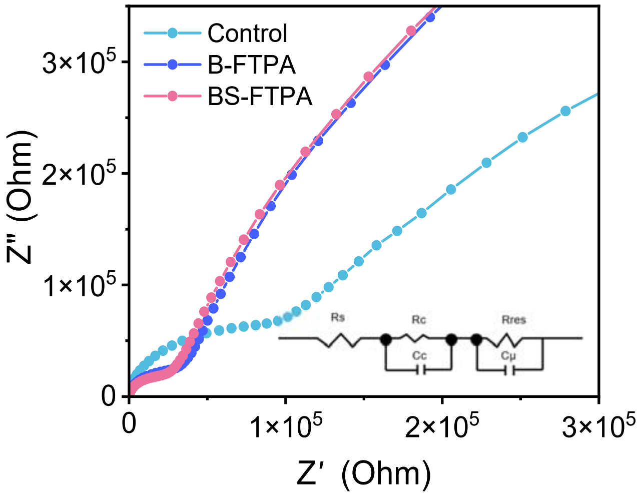


**Supplementary Fig. 23.** Impedance image and the corresponding equivalent circuits of the Control, B-FTPA and BS-FTPA perovskite devices.

**Supplementary Table 2.** Fitted values of different electronic parameters from dark Nyquist plots of Control and FTPA devices.

| Device | $R_s$ (Ω) | $R_{ct}$ (KΩ) | $R_{rec}$ (KΩ) | $C_C$ (nF) | $C_\mu$ (nF) |
|---|---|---|---|---|---|
| Control | 120.08 | 114.00 | 761.85 | 101.67 | 6.98 |
| B-FTPA | 105.00 | 42.87 | 990.85 | 74.44 | 6.84 |
| BS-FTPA | 27.32 | 32.31 | 1130.70 | 80.07 | 6.16 |

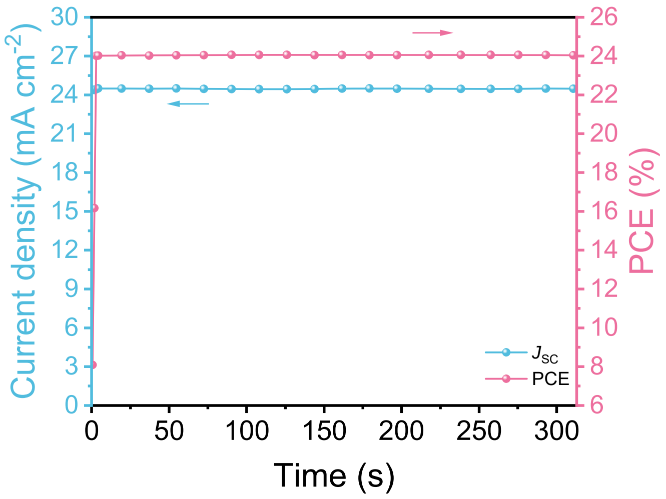


**Supplementary Fig. 24.** Stabilized maximum power output of BS-FTPA at maximum power point as a function of time.

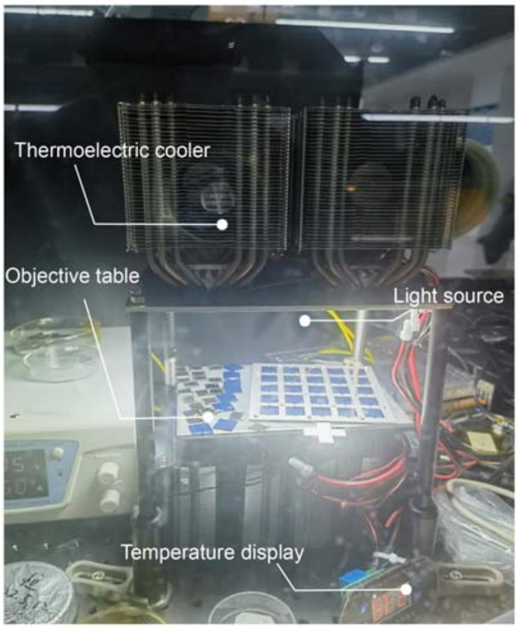


**Supplementary Fig. 25.** For the illumination stability, the test temperature of the PSCs is about 23±2 °C aged in a homemade thermostats instrument combing a thermoelectric cooler, objective table, light source and temperature display.

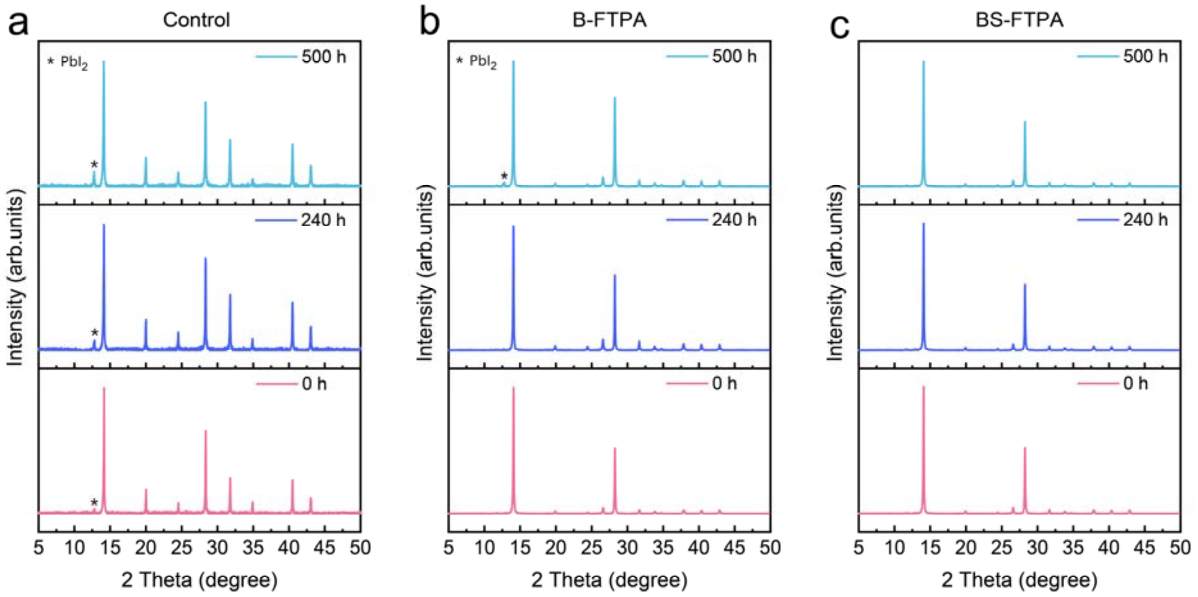


**Supplementary Fig. 26.** Time evolution of XRD patterns of (a) Control, (b) B-FTPA and (c) BS-FTPA perovskite films at temperature of 85 °C under a nitrogen atmosphere.

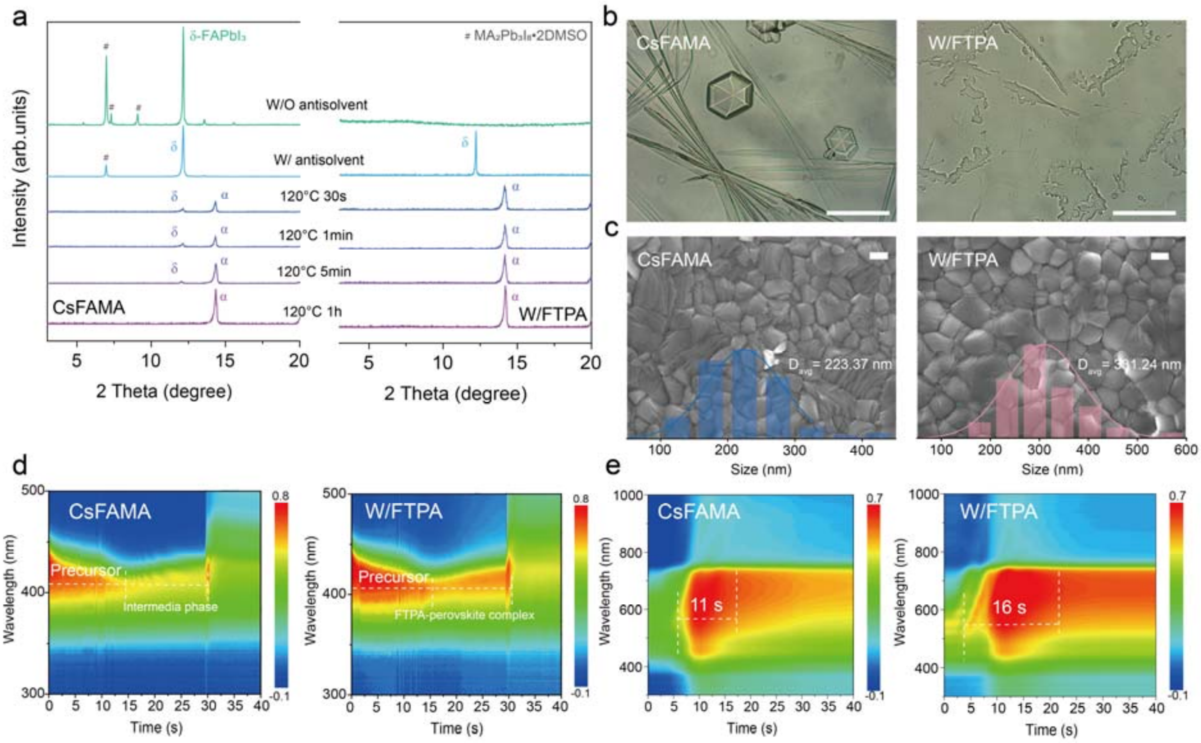


**Supplementary Fig. 27. In-situ monitoring of intermediate phase, nucleation, and crystallization process of the CsFAMA and FTPA based perovskite films during spin-coating and annealing procedures. a**, In-situ tracking of X-ray diffraction of the perovskite films during three processes: wet perovskite film without anti-solvent during spin-coating, wet perovskite film with anti-solvent during spin-coating, and perovskite films annealed at 120 °C for various times. **b**, Optical microscopy images of the wet perovskite films without antisolvent during the spin-coating process. The bar is 50 μm. **c**, Top-view SEM images and distribution histogram of crystal grain sizes of the perovskite thin films. The bar is 200 nm. **d**, The in-situ absorption spectra during the spin-coating process. **e**, In-situ absorption spectra during the initial annealing process at 120 °C.

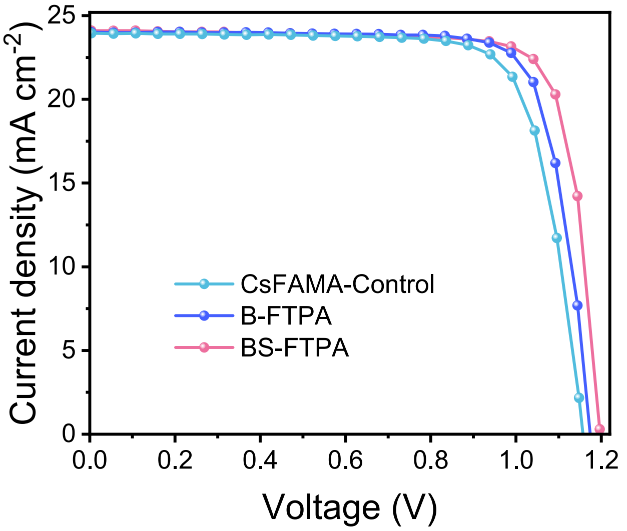


**Supplementary Fig. 28.** Optimal *J-V* curves of (a) $Cs_{0.05}FA_{0.85}MA_{0.10}Pb(I_{0.97}Br_{0.03})_3$, (b) B-FTPA and (c) BS-FTPA PSCs in the reverse scan (RS) direction, respectively. The architecture of the BS-FTPA based PSCs is FTO/CBD-$SnO_2$/perovskite-FTPA/FTPA/Spiro-OMeTAD/Au.

**Supplementary Note 6:** The perovskite precursor solution of $Cs_{0.05}FA_{0.85}MA_{0.10}Pb(I_{0.97}Br_{0.03})_3$ was obtained by dissolving 175.5 mg FAI, 13.1 mg MABr, 16.2 mg CsI and 553.2 mg $PbI_2$ in the 1 mL DMF:DMSO (4:1, v/v) mixed solvent. The device fabrication is the same as FAMA-based PSCs described in the experiment section of the article.

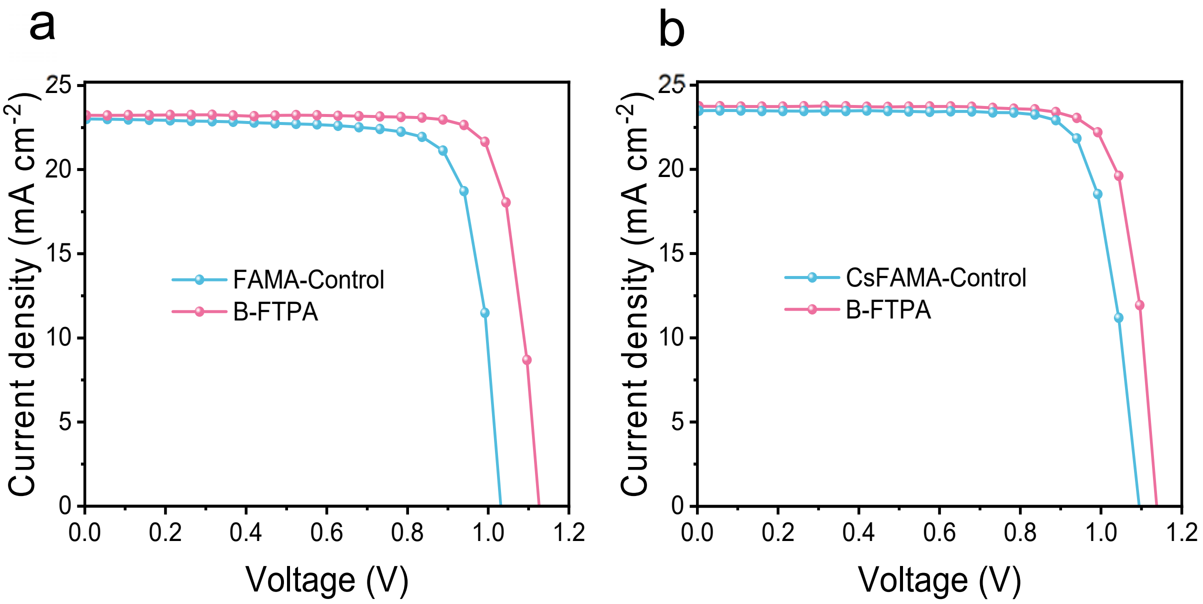


**Supplementary Fig. 29.** *J-V* curves of B-FTPA PSCs using different perovskite precursors, a) $FA_{0.95}MA_{0.05}Pb(I_{0.95}Br_{0.05})_3$ and b) $Cs_{0.05}FA_{0.80}MA_{0.15}Pb(I_{0.85}Br_{0.15})_3$ in the reverse scan direction. The architecture of the PSCs is ITO/PTAA/perovskite (W/FTPA)/$PC_{61}BM$/$C_{60}$/BCP/Ag.

**Supplementary Note 7:** The perovskite precursor preparation of $FA_{0.95}MA_{0.05}Pb(I_{0.95}Br_{0.05})_3$ is in experiment section of the article. The perovskite precursor solution of $Cs_{0.05}FA_{0.80}MA_{0.15}Pb(I_{0.85}Br_{0.15})_3$ was prepared by combining 197.76 mg FAI, 22.38 mg MABr, 17.7 mg CsI, 553.2 mg $PbI_2$ and 73.36 mg $PbBr_2$ in the 0.8 mL DMF:DMSO (4:1, v/v) mixed solvent. Before device fabrication, all the substrates were treated by UVO for 15 min. A 30 µL PTAA solution was spun on the ITO glass substrates at 6000 rpm for 30 s. Then the samples were heated at 100 °C for 10 min. A 60 µL mixed perovskite solution, as well as the B-FTPA perovskite solution (5mol% FTPA addition), was deposited on PTAA substrates by spin-coating at 6000 rpm for 30 s. And 100 µL antisolvent of EA was dropped at the last 10th s and the perovskite films were annealed at 100 °C for 40 min. After that, a 30 µL $PC_{61}BM$ solution was spun on the corresponding perovskite films at 1500 rpm for 45 s. Finally, $C_{60}$ (40 nm)/BCP (5 nm)/Ag (90 nm) was thermally evaporated in sequence. The device contact area was 3.4 mm×3.4 mm.

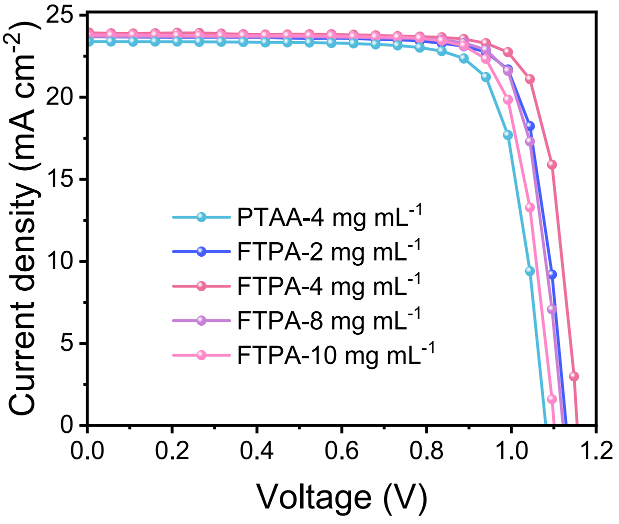


**Supplementary Fig. 30.** Optimal *J-V* curves of the PSCs measured in the reverse scan direction using different concentration of FTPA as HTL and PTAA as reference. The architecture of the PSCs is ITO/PTAA or FTPA/$Cs_{0.05}FA_{0.80}MA_{0.15}Pb(I_{0.85}Br_{0.15})_3$/$PC_{61}BM$/$C_{60}$/BCP/Ag.

**Supplementary Note 8:** Before device fabrication, all the substrates were treated by UVO for 15 min. A 30 μL FTPA solution was spun on the ITO glass substrates at 6000 rpm for 30 s. Then the samples were heated at 100 °C for 20 min, respectively. A 60 μL mixed perovskite solution was deposited on FTPA substrates by spin-coating at 6000 rpm for 30 s. And 100 μL antisolvent of EA was dropped at the last 10s and the perovskite films were annealed at 100 °C for 40 min. After that, a 30 μL $PC_{61}BM$ solution was spun on the corresponding perovskite films at 1500 rpm for 45 s. Finally, $C_{60}$ (40 nm)/BCP (5 nm)/Ag (90 nm) was thermally evaporated in sequence. The device contact area was 3.4 mm×3.4 mm.

**Supplementary Table 3.** The best photovoltaic parameters of PSCs using different concentration of FTPA as HTL and PTAA as reference measured in reverse scan directions under standard AM 1.5 illumination (100 mW $cm^{-2}$).

| | Concentration ($mg\ mL^{-1}$) | $V_{OC}$ (V) | $J_{SC}$ ($mA\ cm^{-2}$) | FF (%) | PCE (%) |
|---|---|---|---|---|---|
| PTAA | 4 | 1.081 | 23.39 | 78.89 | 19.95 |
| FTPA | 2 | 1.130 | 23.70 | 80.41 | 21.53 |
| | 4 | 1.156 | 23.93 | 81.27 | 22.48 |
| | 8 | 1.122 | 23.71 | 80.21 | 21.34 |
| | 10 | 1.101 | 23.78 | 80.03 | 20.95 |

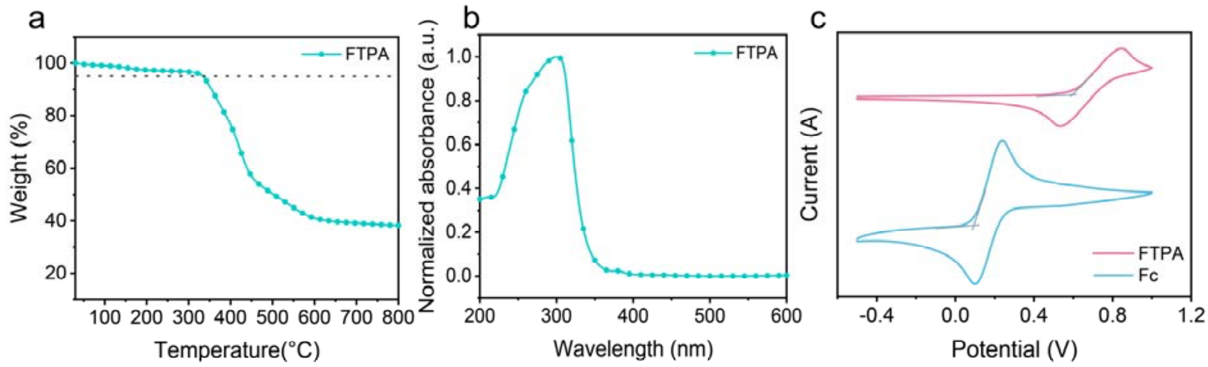


**Supplementary Fig. 31.** Characterizations of Molecular FTPA. Thermal analysis. (a) TGA curves of FTPA, (b) UV-vis absorption spectra of the FTPA film, (c) Cyclic voltammogram of the FTPA and Fc measured at room temperature in a 0.1 mol $L^{-1}$ $Bu_4NPF_6$-$CH_2Cl_2$ solution, and the scan rate was 100 $mV{\cdot}s^{-1}$.

**Supplementary Table 4.** Detailed molecular and material parameters on the optical, electrochemical of the synthesized FTPA.

| | $T_d$ (°C) | $T_c$[a] (°C) | $T_c$[b] (°C) | $\lambda_{abs}$ (nm) | $E_g$[c] (eV) | $E_{HOMO}$ (eV) | $E_{LUMO}$ (eV) |
|---|---|---|---|---|---|---|---|
| FTPA | 332.5 | 158.0 | 100.0 | 360.0 | 3.40 | -5.30 | -1.90 |

Crosslinking temperature of [a] pure FTPA and [b] FTPA (with AIBN) measured by differential scanning calorimetry (DSC) [c] Optical band gap ($E_g$) was calculated from the equation, $E_g$=(1240/$\lambda_{onset}$), where $\lambda_{onset}$ the absorption onset wavelengths from the UV-Vis spectra of FTPA, [d] First oxidation potential measured by solution-based cyclic voltammetry (CV), [e] LUMO calculated by LUMO = HOMO + $E_g$.

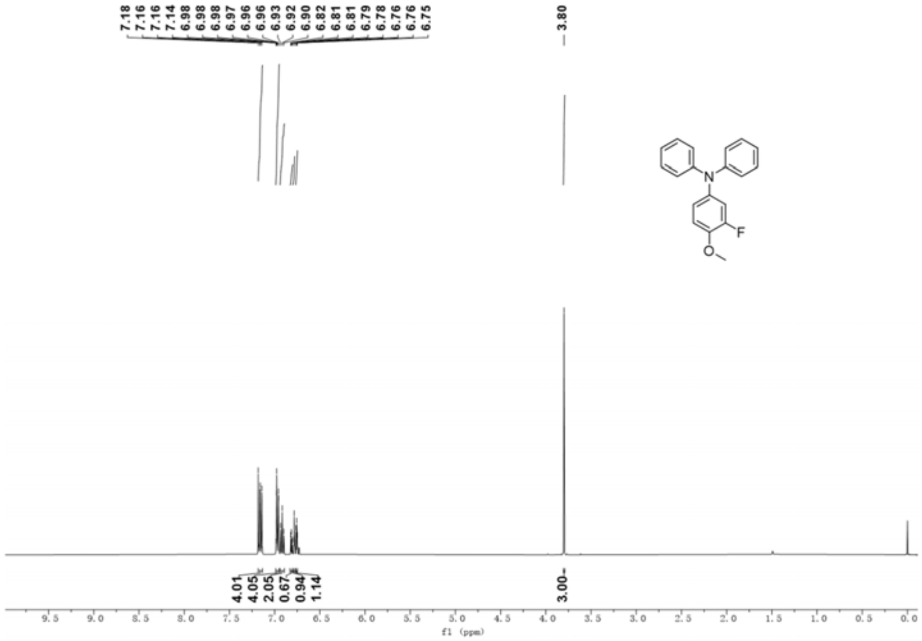


**Supplementary Fig. 32.** $^{1}H$ NMR spectrum of 3-fluoro-4-methoxy-N,N-diphenylaniline (Compound **1**).

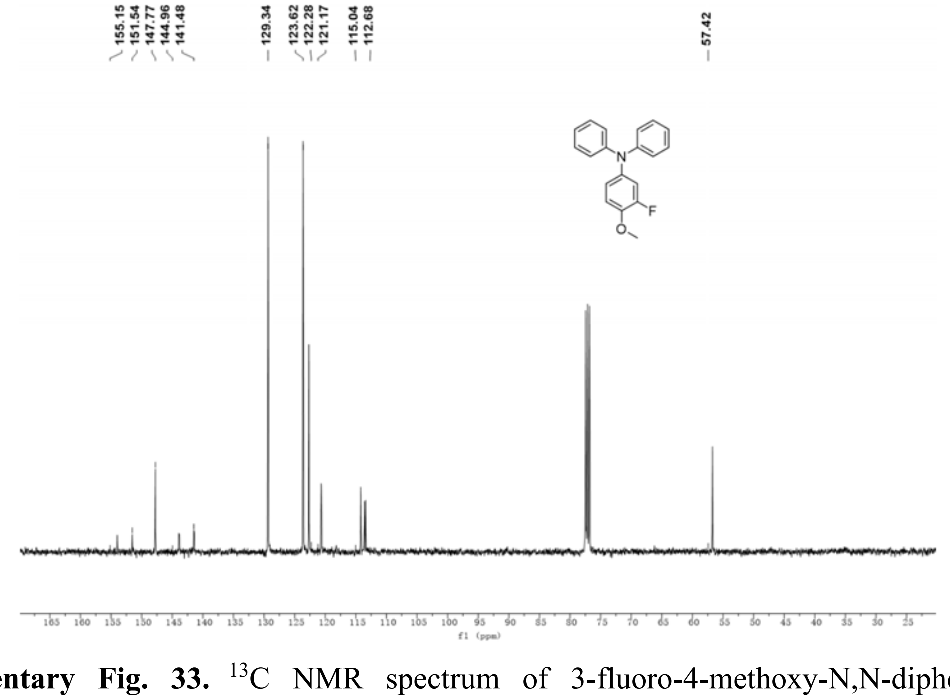


**Supplementary Fig. 33.** $^{13}C$ NMR spectrum of 3-fluoro-4-methoxy-N,N-diphenylaniline (Compound **1**).

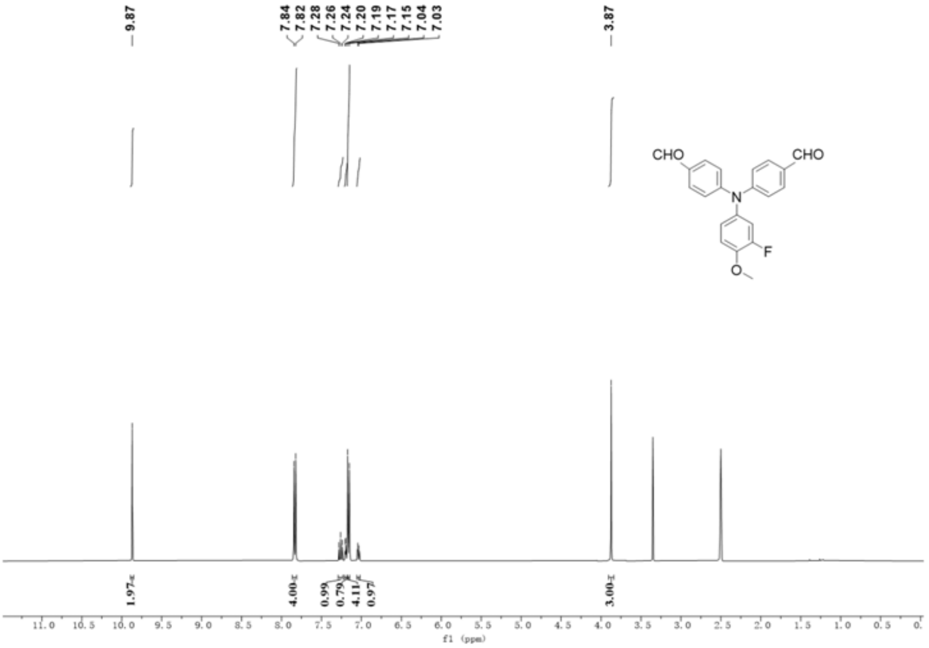


**Supplementary Fig. 34.** $^{1}H$ NMR spectrum of 4,4'-((3-fluoro-4-methoxyphenyl)azanediyl)dibenzaldehyde (Compound **2**).

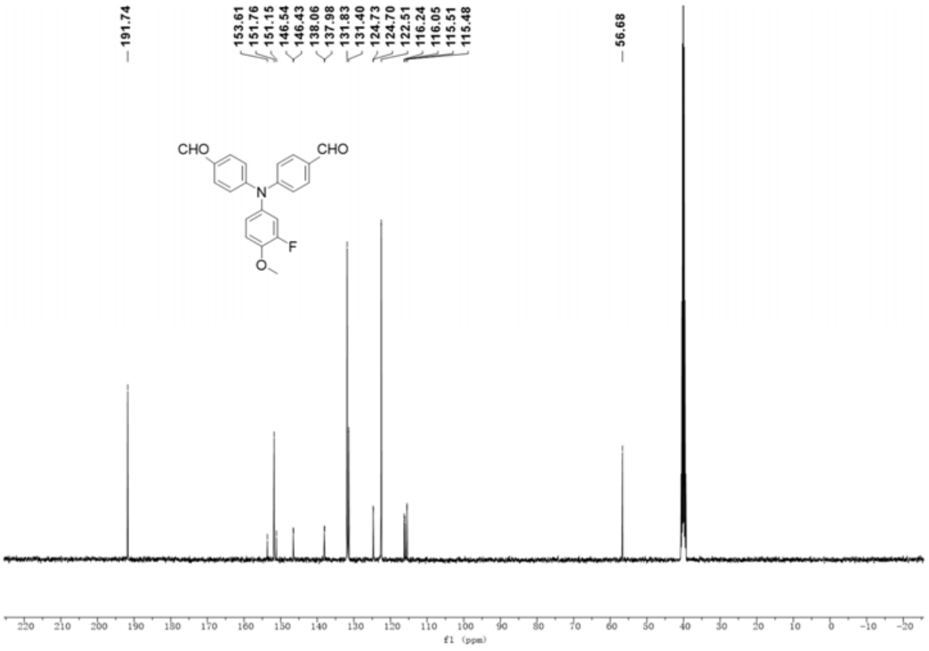


**Supplementary Fig. 35.** $^{13}C$ NMR spectrum of 4,4'-((3-fluoro-4-methoxyphenyl)azanediyl)dibenzaldehyde (Compound **2**).

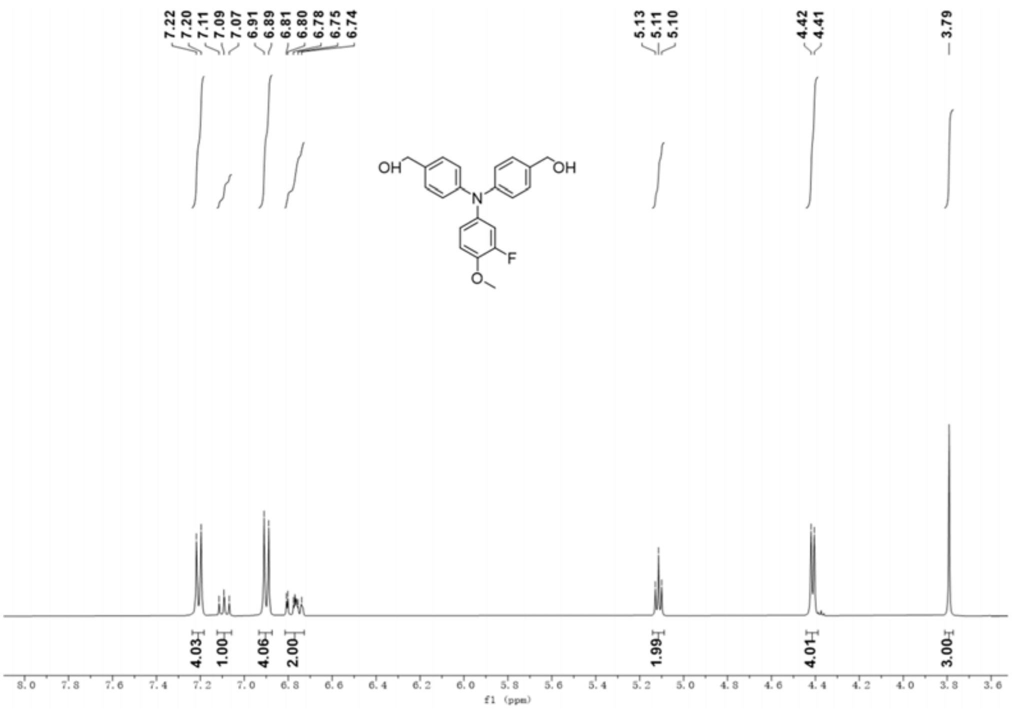

**Supplementary Fig. 36.** $^{1}$H NMR spectrum of (((3-fluoro-4-methoxyphenyl)azanediyl)bis(4,1-phenylene))dimethanol (Compound **3**).

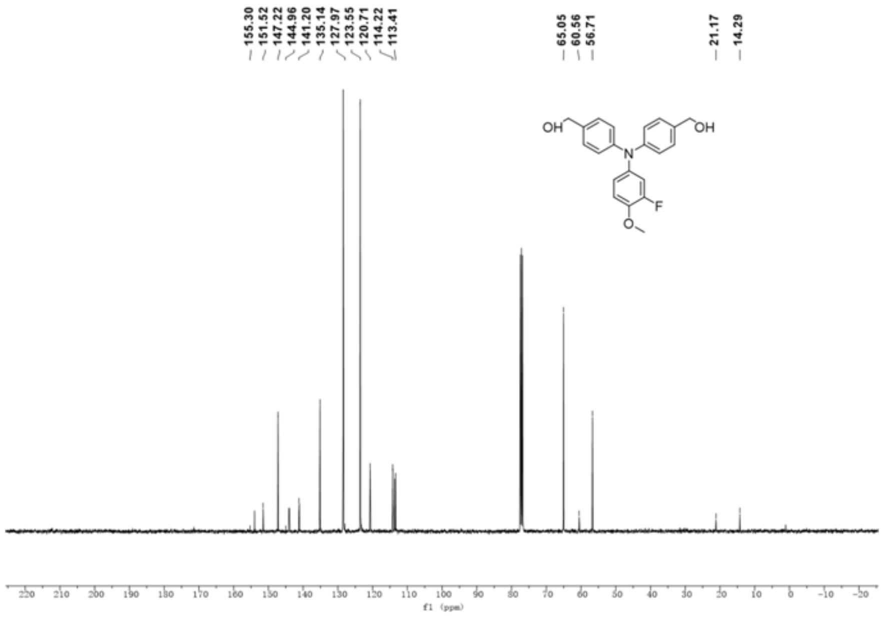

**Supplementary Fig. 37.** $^{13}$C NMR spectrum of (((3-fluoro-4-methoxyphenyl)azanediyl)bis(4,1-phenylene))dimethanol (Compound **3**).

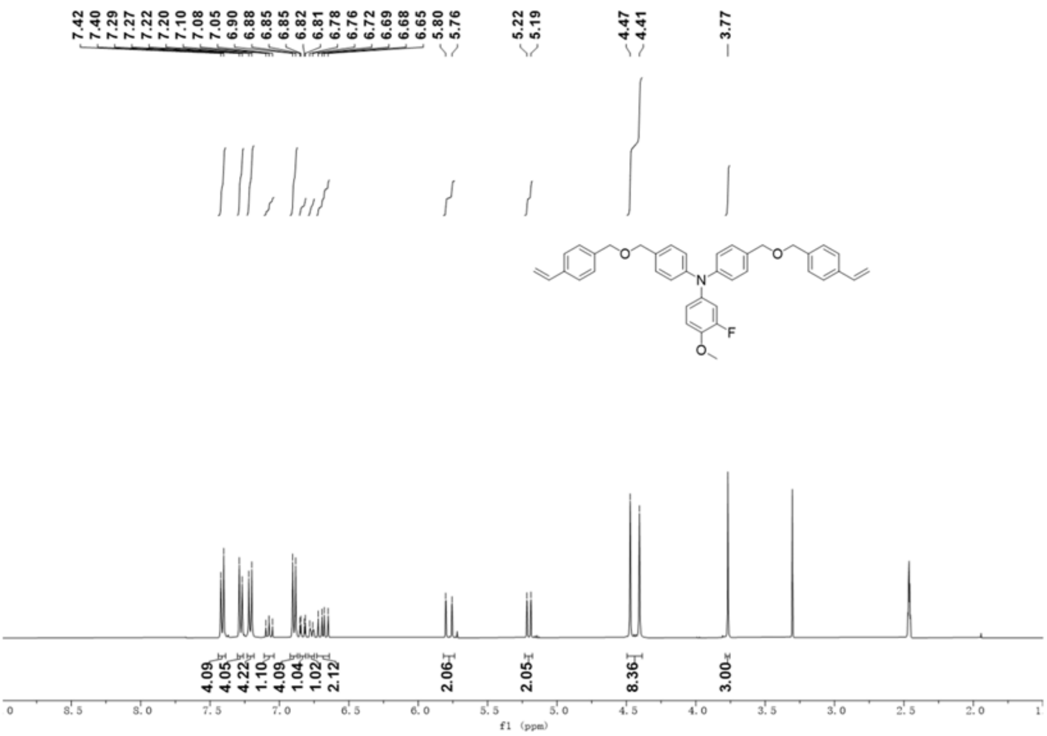


**Supplementary Fig. 38.** $^{1}$H NMR spectrum of 3-fluoro-4-methoxy-4',4''-bis((4-vinylbenzyl ether) methyl)) triphenylamine (FTPA).

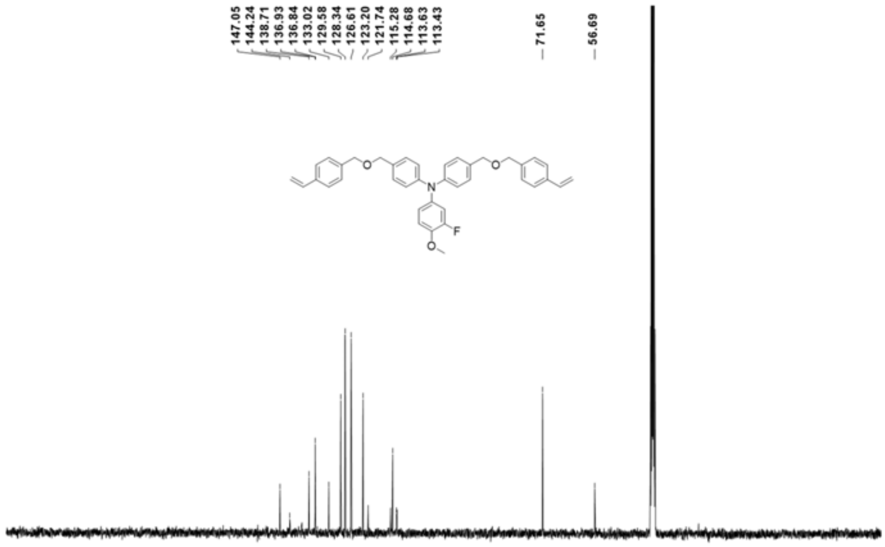


**Supplementary Fig. 39.** $^{13}$C NMR spectrum of 3-fluoro-4-methoxy-4',4''-bis((4-vinylbenzyl ether) methyl)) triphenylamine (FTPA).

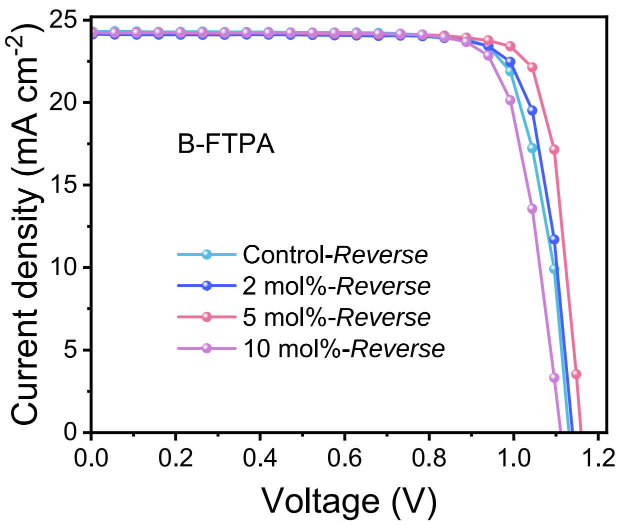


**Supplementary Fig. 40.** Optimal *J*-*V* curves of the n-i-p PSCs with different FTPA additive concentration in the bulk of perovskite measured in the reverse scan direction.

**Supplementary Table 5.** The best photovoltaic parameters of n-i-p PSCs for the control and B-FTPA devices with different FTPA concentration measured in reverse scan directions under standard AM 1.5 illumination (100 mW cm$^{-2}$).

| | FTPA (mol%) | $V_{OC}$ (V) | $J_{SC}$ (mA cm$^{-2}$) | FF (%) | PCE (%) |
|---|---|---|---|---|---|
| B-FTPA | Control | 1.131 | 24.29 | 80.11 | 22.01 |
| | 2 (17.25 mg mL$^{-1}$) | 1.139 | 24.13 | 81.08 | 22.29 |
| | 5 (43.13 mg mL$^{-1}$) | 1.161 | 24.25 | 82.41 | 23.22 |
| | 10 (86.26 mg mL$^{-1}$) | 1.113 | 24.23 | 79.64 | 21.48 |

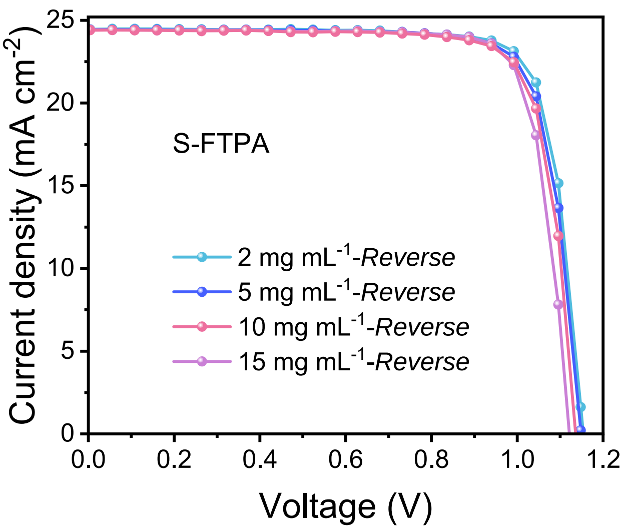


**Supplementary Fig. 41.** Optimal *J-V* curves of the n-i-p PSCs with different FTPA concentration on the surface of the perovskite measured in the reverse scan direction.

**Supplementary Table 6.** The best photovoltaic parameters of PSCs for the n-i-p PSCs with different FTPA concentration measured in reverse scan directions under standard AM 1.5 illumination (100 mW cm$^{-2}$).

| | FTPA (mg mL$^{-1}$) | $V_{OC}$ (V) | $J_{SC}$ (mA cm$^{-2}$) | FF (%) | PCE (%) |
|---|---|---|---|---|---|
| S-FTPA | 2 | 1.154 | 24.46 | 81.68 | 23.05 |
| | 5 | 1.148 | 24.44 | 80.50 | 22.58 |
| | 10 | 1.136 | 24.40 | 80.37 | 22.27 |
| | 15 | 1.122 | 24.43 | 79.91 | 21.90 |

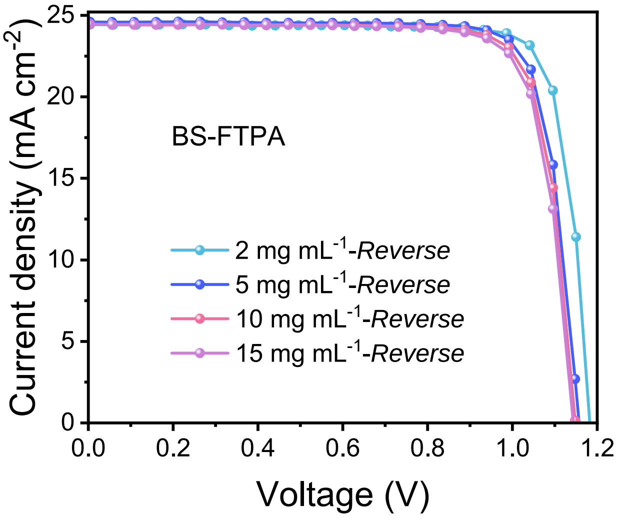


**Supplementary Fig. 42.** Optimal *J-V* curves of the B-FTPA (5mol% FTPA in bulk) based n-i-p PSCs with different FTPA interfacial treatment concentration (mg $mL^{-1}$) measured in the reverse scan direction.

**Supplementary Table 7.** The best photovoltaic parameters of PSCs for the B-FTPA (5 mol% FTPA in bulk) based n-i-p PSCs with different FTPA interfacial treatment concentration measured in reverse scan directions under standard AM 1.5 illumination (100 mW $cm^{-2}$).

| | FTPA (mg $mL^{-1}$) | $V_{OC}$ (V) | $J_{SC}$ (mA $cm^{-2}$) | FF (%) | PCE (%) |
|---|---|---|---|---|---|
| BS-FTPA | 2 | 1.182 | 24.43 | 83.45 | 24.10 |
| | 5 | 1.158 | 24.59 | 81.84 | 23.32 |
| | 10 | 1.148 | 24.45 | 81.37 | 22.85 |
| | 15 | 1.143 | 24.46 | 80.44 | 22.49 |

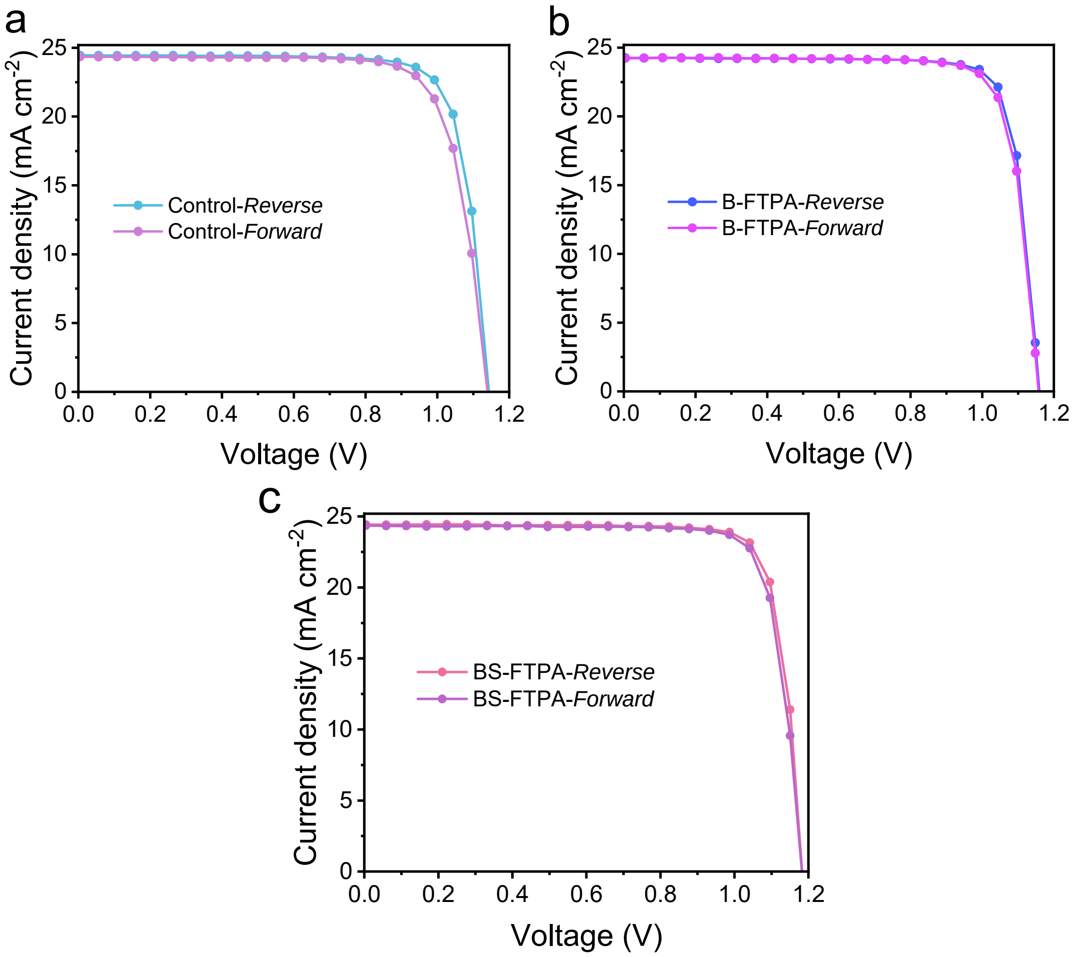


**Supplementary Fig. 43.** *J-V* curves of (a) Control, (b) B-FTPA and (c) BS-FTPA PSCs in a reverse scan (RS) and forward scan (FS), respectively.

**Supplementary Table 8.** Summary of photovoltaic parameters of Control, B-FTPA and BS-FTPA PSCs in a reverse scan (RS) and forward scan (FS), respectively.

| | Scan Directions | $V_{OC}$ (V) | $J_{SC}$ (mA cm$^{-2}$) | FF (%) | PCE (%) |
|---|---|---|---|---|---|
| Control | RS | 1.143 | 24.46 | 80.38 | 22.48 |
| | FS | 1.139 | 24.35 | 77.77 | 21.51 |
| B-FTPA | RS | 1.161 | 24.25 | 82.41 | 23.22 |
| | FS | 1.158 | 24.23 | 81.64 | 22.92 |
| BS-FTPA | RS | 1.182 | 24.43 | 83.45 | 24.10 |
| | FS | 1.182 | 24.36 | 82.31 | 23.70 |

DFT calculation

Theoretical calculations were carried out with a Gaussian 09 D.01 package using b3lyp/6-31g(d, p) method.[4]

**Supplementary References**


1. Zhao, Y. et al. A Polymerization-assisted grain growth strategy for efficient and stable perovskite solar cells. *Adv. Mater.* **32**, 1907769 (2020).

2. Guo, S., Wan, W., Chen, C. & W. H. Chen. Thermal decomposition kinetic evaluation and its thermal hazards prediction of AIBN. *J. Therm. Anal. Calorim.***113**, 1169–1176 (2013).

3. Li, X. R., Wang, X. L. & Koseki, H. Study on thermal decomposition characteristics of AIBN. *J. Hazard. Mater.* **159**, 13 (2008)

4. Frisch, M. J., et al. Gaussian 16, Revision A.03: Gaussian, Inc., Wallingford CT, (2016).
