# Supplementary material for: Orientated crystallization of FA-based perovskite via hydrogen-bonded polymer network for efficient and stable solar cells
Source: Nat Commun. 2023 Feb 2;14:573. doi: 10.1038/s41467-023-36224-6 (PMC9895431; doi:10.1038/s41467-023-36224-6)
Supplement: Supplementary file 3 — Description of Additional Supplementary Files [file 41467_2023_36224_MOESM3_ESM.pdf]

Description of Additional Supplementary Files

File Name: Supplementary Movie 1

Description: Accelerated Video of control and BS-FTPA perovskite films immersed into the water during 10 minutes at R.T.
