# Supplementary material for: Orientated crystallization of FA-based perovskite via hydrogen-bonded polymer network for efficient and stable solar cells
Source: Nat Commun. 2023 Feb 2;14:573. doi: 10.1038/s41467-023-36224-6 (PMC9895431; doi:10.1038/s41467-023-36224-6)
Supplement: Supplementary file 5 — Solar Cells Reporting Summary [file 41467_2023_36224_MOESM5_ESM.pdf]

nature research

Corresponding author(s): Fangfang Wang, Wei Huang, Tianshi Qin

Solar Cells Reporting Summary

Nature Research wishes to improve the reproducibility of the work that we publish. This form is intended for publication with all accepted papers reporting the characterization of photovoltaic devices and provides structure for consistency and transparency in reporting. Some list items might not apply to an individual manuscript, but all fields must be completed for clarity.

For further information on Nature Research policies, including our data availability policy, see Authors & Referees.

Experimental design

Please check: are the following details reported in the manuscript?

1. Dimensions

Item	Yes/No	Response
Area of the tested solar cells	☒ Yes ☐ No	The device area is 0.1 cm^2.
Method used to determine the device area	☒ Yes ☐ No	The metal mask.

2. Current-voltage characterization

Item	Yes/No	Response
Current density-voltage (J-V) plots in both forward and backward direction	☒ Yes ☐ No	The (J-V) polts are provided in the manuscript .
Voltage scan conditions<br>*For instance: scan direction, speed, dwell times*	☒ Yes ☐ No	The bias ranges is from -0.1 to 1.3 V under reverse and forward voltage scan. Speed: 233mV/s, Dwell time: 0.1s
Test environment<br>*For instance: characterization temperature, in air or in glove box*	☒ Yes ☐ No	All J-V results are measured at 25±2°C in glove box.
Protocol for preconditioning of the device before its characterization	☐ Yes ☒ No	No precondition is used in this work.
Stability of the J-V characteristic<br>*Verified with time evolution of the maximum power point or with the photocurrent at maximum power point; see ref. 7 for details.*	☒ Yes ☐ No	Time evolution of the maximum power point and the photocurrent at maximum power point are provided in Supplementary Fig. 24 .

3. Hysteresis or any other unusual behaviour

Item	Yes/No	Response
Description of the unusual behaviour observed during the characterization	☒ Yes ☐ No	Very minor hysteresis for the devices.
Related experimental data	☒ Yes ☐ No	J-V plots under reverse and forward are provided in Supplementary Fig. 43. and Supplementary Table 8.

4. Efficiency

Item	Yes/No	Response
External quantum efficiency (EQE) or incident photons to current efficiency (IPCE)	☒ Yes ☐ No	IPCE are provided in Fig. 4c in the manuscript.
A comparison between the integrated response under the standard reference spectrum and the response measure under the simulator	☒ Yes ☐ No	IPCE spectra demonstrated matchable integrated JSC values (<5% deviation) to J-V scan data.
For tandem solar cells, the bias illumination and bias voltage used for each subcell	☐ Yes ☒ No	Tandem solar cells are not covered in this paper.

5. Calibration

Item	Yes/No	Response
Light source and reference cell or sensor used for the characterization	☒ Yes ☐ No	Current density-voltage (J–V) curves were measured using a solar simulator (Class 3A, XES-40S3, SAN-EI) at AM1.5G illumination equipped with a Keithley 2400 source meter. The standard silicon solar cell calibrated by Newport was used to calibrate the light intensity to AM1.5G one sun (100 mW cm^-2).

Confirmation that the reference cell was calibrated and certified	☒ Yes ☐ No	The standard silicon solar cell calibrated by Newport was used to calibrate the light intensity to AM1.5G one sun (100 mW cm^-2).
Calculation of spectral mismatch between the reference cell and the devices under test	☐ Yes ☒ No	The standard silicon solar cell calibrated by Newport was used to calibrate the light intensity to AM1.5G one sun (100 mW cm^-2).

6. Mask/aperture

Size of the mask/aperture used during testing	☒ Yes ☐ No	Metal mask with area of 0.1 cm^2.
Variation of the measured short-circuit current density with the mask/aperture area	☐ Yes ☒ No	All J-V results are measured with mask.

7. Performance certification

Identity of the independent certification laboratory that confirmed the photovoltaic performance	☐ Yes ☒ No	The solar cells efficiency is not certified.
A copy of any certificate(s) *Provide in Supplementary Information*	☐ Yes ☒ No	No certified.

8. Statistics

Number of solar cells tested	☒ Yes ☐ No	30 solar cells were tested.
Statistical analysis of the device performance	☒ Yes ☐ No	This has been stated in the manuscript in Fig. 4e.

9. Long-term stability analysis

Type of analysis, bias conditions and environmental conditions *For instance: illumination type, temperature, atmosphere humidity, encapsulation method, preconditioning temperature*	☒ Yes ☐ No	Figure 4f. Device stability of unencapsulated devices under 1-sun illumination at 23 ±2 °C in a nitrogen atmosphere (ISOS-LC-1). g, Device stability of unencapsulated devices held at 25±10 °C and 50H±10% relative humidity (ISOS-D-1). i, Device stability of unencapsulated devices under 65±3°C thermal aging (ISOS-T-1). All of the error bars represent the standard deviation for six devices.
